# Selective requirement for polycomb repressor complex 2 in the generation of specific hypothalamic neuronal subtypes

**DOI:** 10.1242/dev.200076

**Published:** 2022-03-07

**Authors:** Behzad Yaghmaeian Salmani, Brad Balderson, Susanne Bauer, Helen Ekman, Annika Starkenberg, Thomas Perlmann, Michael Piper, Mikael Bodén, Stefan Thor

**Affiliations:** 1 Department of Clinical and Experimental Medicine, Linkoping University, SE-58185 Linkoping, Sweden; 2 Department of Cell and Molecular Biology, Karolinska Institute, SE-17177 Stockholm, Sweden; 3 School of Chemistry and Molecular Biosciences, University of Queensland, St Lucia, QLD 4072, Australia; 4 School of Biomedical Sciences, University of Queensland, St Lucia, QLD 4072, Australia

**Keywords:** Cell specification, Epigenetics, H3K27me3, H3K4me1/3, Neuropeptide neurons, Mouse

## Abstract

The hypothalamus displays staggering cellular diversity, chiefly established during embryogenesis by the interplay of several signalling pathways and a battery of transcription factors. However, the contribution of epigenetic cues to hypothalamus development remains unclear. We mutated the polycomb repressor complex 2 gene *Eed* in the developing mouse hypothalamus, which resulted in the loss of H3K27me3, a fundamental epigenetic repressor mark. This triggered ectopic expression of posteriorly expressed regulators (e.g. Hox homeotic genes), upregulation of cell cycle inhibitors and reduced proliferation. Surprisingly, despite these effects, single cell transcriptomic analysis revealed that most neuronal subtypes were still generated in *Eed* mutants. However, we observed an increase in glutamatergic/GABAergic double-positive cells, as well as loss/reduction of dopamine, hypocretin and Tac2-Pax6 neurons. These findings indicate that many aspects of the hypothalamic gene regulatory flow can proceed without the key H3K27me3 epigenetic repressor mark, but points to a unique sensitivity of particular neuronal subtypes to a disrupted epigenomic landscape.

## Abstract (precis)

**Summary:** Polycomb repressor complex 2 inactivation results in selective effects on mouse hypothalamic development, increasing glutamatergic/GABA cells, while reducing dopamine, Hcrt and Tac2-Pax6 cells.

## INTRODUCTION

The hypothalamus acts as a master homeostatic regulator, controlling energy and fluid balance, thermoregulation, sleep-wake states, stress responses, growth and reproduction, as well as emotional and social behaviours (Saper and Lowell, 2014). The hypothalamus can perform this plethora of complex functions in large part due to its staggering neuronal diversity (Alpár et al., 2019; Romanov et al., 2019). Microarray and single cell transcriptomic analyses of the adult hypothalamus have resulted in major leaps in our understanding of the complete mature cellular diversity in this tissue, pointing to the presence of ∼50 major cell types and several hundred subtypes (Campbell et al., 2017; Chen et al., 2017; Dalal et al., 2013; Henry et al., 2015; Jeong et al., 2016; Kim et al., 2019; Kurrasch et al., 2007; Lam et al., 2017; Mickelsen et al., 2019, 2017; Moffitt et al., 2018; Romanov et al., 2017; Shimogori et al., 2010).

The development of the hypothalamus has been challenging to decode, not only because of its immense cellular diversity. Unlike other CNS regions, such as the cortex, hindbrain or spinal cord, which are arranged in columnar structures, the adult hypothalamus is characterized by a patchwork of partially overlapping nuclei and territories. Hypothalamic development is also characterized by complex tissue rearrangements and cellular migration (Bedont et al., 2015; Burbridge et al., 2016; Ferran et al., 2015; Puelles and Rubenstein, 2015). Despite these challenges, extensive efforts have resulted in the unravelling of a multi-step process of anterior-posterior and medio-lateral patterning events, involving many of the major signalling pathways (Shh, BMP, Nodal, WNT and FGF). This patterning process results in, and integrates with, the selective expression of a number of early transcription factors (TFs), which act to further subdivide the hypothalamus. These early TFs in turn activate panels of late TFs within subdomains of the developing hypothalamus, which act with more restrictive mandates to specify diverse subsets of hypothalamic cell fates (Alvarez-Bolado, 2019; Bedont et al., 2015; Blackshaw et al., 2010; Burbridge et al., 2016; Ferran et al., 2015; Nesan and Kurrasch, 2016; Puelles and Rubenstein, 2015; Xie and Dorsky, 2017). More recently, single cell transcriptomic analysis of the embryonic hypothalamus has greatly increased our understanding of its developmental process (Huisman et al., 2019; Kim et al., 2020; Romanov et al., 2020; Zhang et al., 2021b; Zhou et al., 2020).

In contrast to the identification of key signalling cues and TF pathways, the role of epigenetics in the control of hypothalamus development is not well understood. An intensively studied epigenetic regulator is the polycomb group complex (PcG), which is a collective name that refers to several different subcomplexes, where the polycomb repressor complexes 1 and 2 (PRC1 and PRC2) are arguably most well-defined (Piunti and Shilatifard, 2016; Steffen and Ringrose, 2014). PRC2 mono-, di- and tri-methylates residue K27, chiefly on histone 3.3 and, to a lesser extent H3.2 and H3.1 (Banaszynski et al., 2013). In general, PRC2 triggers transcriptional repression of target genes, although its specific role in gene regulation and chromatin compaction is still under intensive investigation, an issue that is further challenged by the existence of variations in PRC2 protein complex composition (Chammas et al., 2020; Laugesen et al., 2019; van Mierlo et al., 2019). In mammals, the embryonic ectoderm development (*Eed*) gene constitutes a crucial component of PRC2 and is encoded by a single gene in the mouse genome. *Eed* null mutants display an apparently complete loss of H3K27me1/2/3 (Montgomery et al., 2005), and when *Eed* is selectively removed in, for example, the haematopoietic lineage, in the intestine or in the CNS, H3K27me3 is lost (Jadhav et al., 2020; Xie et al., 2014; Yaghmaeian Salmani et al., 2018). Hence, in contrast to most, if not all, other epigenetic marks and their enzyme systems, the single gene removal of *Eed* provides an unparalleled means of completely removing this key epigenetic mark.

To begin addressing the role of epigenetic input on hypothalamic development, we analyzed *Eed* conditional mutants (*Eed-cKO*), where *Eed* was deleted by the early CNS deleter *Sox1-Cre*, which is active at embryonic day (E) 8.5 (Takashima et al., 2007). We found that *Eed-cKO* mutants display a complete loss of H3K27me3 in the hypothalamus, from E11.5 and onwards. *Eed-cKO* mutants upregulate several cell cycle inhibitor genes and display reduced proliferation in the hypothalamus. We also observed ectopic expression of many posteriorly expressed TFs, indicating posteriorization of the anterior CNS. To unravel the effects of *Eed-cKO* upon cell specification, we conducted single cell transcriptomic (scRNA-seq) analysis at E13.5, E15.5 and E18.5, spanning the major phase of hypothalamic cell specification (Kim et al., 2020; Romanov et al., 2020). Surprisingly, despite reduced proliferation and extensive ectopic TF expression, scRNA-seq analysis revealed that most hypothalamic subtypes were generated in the *Eed-cKO* mutants. However, there was an increase in glutamatergic/GABAergic double-positive cells, as well as loss/reduction of dopamine, hypocretin (orexin), and a subgroup of Tac2 cells (Tac2-Pax6). scRNA-seq analysis revealed that these effects may result from dysregulation of several known cell-fate determinants. These findings suggest that many, but not all, aspects of the gene regulatory pathways necessary for hypothalamic development can play out irrespective of the H3K27me3 epigenetic mark, but point to higher sensitivity of certain neuronal subtypes to an altered epigenomic landscape.

## RESULTS

### Conditional knockout of *Eed* in the CNS results in the loss of H3K27me3

Constitutive *Eed* mutants die during early embryogenesis (Faust et al., 1998, 1995; Schumacher et al., 1996). To circumvent this early lethality we knocked out *Eed* conditionally in the CNS, by crossing a previously generated floxed *Eed* allele (Xie et al., 2014) to *Sox1-Cre* (Takashima et al., 2007). *Sox1* is expressed in the entire CNS and commences in the neural plate at E7.5 (Pevny et al., 1998). *Sox1-Cre* is a *Cre* insertion into the *Sox1* locus, and expresses *Cre* in agreement with the *Sox1* gene, as evident by Cre-mediated activation of a *ROSA26R-EYFP* reporter strain at E8.5 in the entire developing CNS (Takashima et al., 2007). In contrast to *Eed* constitutive mutants, our conditional *Eed* mutants (denoted *Eed-cKO*) developed until at least E18.5. Hence, deleting *Eed* using *Sox1-Cre* circumvents early lethality while specifically removing *Eed* function from the entire developing hypothalamus at the earliest possible stage. We previously found that *Eed-cKO* embryos displayed loss of H3K27me3 immunostaining in the dorsal telencephalon and lumbo-sacral spinal cord, with a reduction of immunostaining at E10.5 and a complete loss at E11.5, and displayed an undergrown brain, in particular of the telencephalon (Yaghmaeian Salmani et al., 2018). Focusing upon the developing hypothalamus we observed a loss of H3K27me3 immunostaining in the hypothalamus in *Eed-cKO* mutants, at E11.5 (Fig. S1A-J). Based upon these findings, and our previous study, we conclude that deletion of *Eed* by the early CNS-specific deleter *Sox1-Cre* results in loss of the H3K27me3 mark, albeit with a 2-3-day delay.

### *Eed-cKO* mutants display reduced hypothalamic proliferation

*Eed-cKO* mutants display reduced proliferation in the dorsal telencephalon and an undergrown telencephalon, whereas the lumbo-sacral spinal cord did not display any apparent change in proliferation (Yaghmaeian Salmani et al., 2018). To address proliferation in the hypothalamus, we used the CNS progenitor marker Sox2, phosphorylated Ser-28 on Histone 3 (PH3, a marker of mitotic cells), and 4′,6-diamidino-2-phenylindole (DAPI) nuclear staining, to assess total nuclear (cellular) volume. Focusing on E15.5, in the *Eed-cKO* mutants we observed fewer PH3+ cells in the hypothalamus than in control (*Eed^fl/fl^*) (Fig. S2A-H). Quantification supported this notion and revealed significantly fewer PH3+ cells/DAPI volume and fewer PH3+ cells/Sox2 volume (Fig. S2I,J). We did not however observe reduction of the percentage of Sox2-expressing cells/DAPI volume (Fig. S2K). Therefore, similar to our previous findings for the telencephalon (Yaghmaeian Salmani et al., 2018), *Eed-cKO* mutants displayed reduced proliferation in the hypothalamus during embryogenesis.

### Single cell transcriptional profiling reveals upregulation of H3K27me3 marked genes in *Eed* mutants

To address the effects of *Eed* mutation upon hypothalamus development in more detail we performed scRNA-seq analysis. We focused first upon a late embryonic stage, E18.5, because the majority of distinct neuronal subtypes – such as those specifically expressing neuropeptides and neurotransmitters – have been generated by this stage (Kim et al., 2020; Romanov et al., 2020; Zhang et al., 2021b). We dissected the hypothalamus from six control (*Eed^fl/fl^*) and nine *Eed-cKO* embryos at E18.5 (two female and four male embryos for control; five female and four male embryos for *Eed-cKO*). Cells were dissociated and sequenced on the Illumina/Bio-Rad ddSEQ platform. We analyzed 48 individual samples (seven female and 15 male samples for control; 15 female and 11 male samples for *Eed-cKO*), yielding a total of 20,703 cells.

**Fig. 1.**
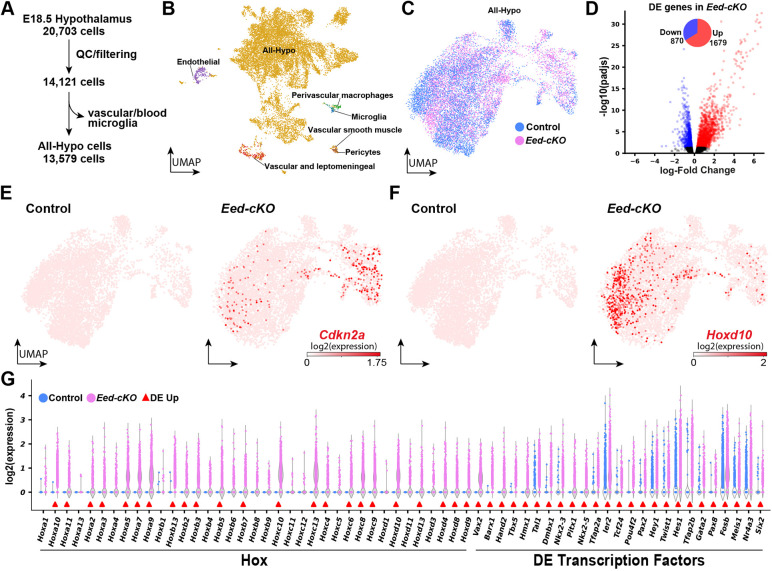
**Single cell transcriptomic analysis of *Eed-cKO* mutants reveals upregulation of TFs and Hox genes in the hypothalamus.** (A) Process for quality control (QC) and biological filtering of hypothalamic scRNA-seq cells. (B) UMAP of hypothalamic scRNA-seq cells, combined from control and *Eed-cKO*, at E18.5, based upon 300 genes (Table S1). (C) UMAP of All-Hypo cells in control and *Eed-cKO*. (D) Volcano plot showing DEGs in *Eed-cKO*. (E,F) UMAP of E18.5 hypothalamic scRNA-seq All-Hypo cells, showing expression of *Cdkn2a* (E) and *Hoxd10* (F), in control and *Eed-cKO*. (G) Violin plots comparing the expression levels of Hox genes and the highest differentially expressed (DE) transcription factors between control and *Eed-cKO* in E18.5 hypothalamic scRNA-seq All-Hypo cells (adjusted *P*-value≤0.05).

Based upon the distribution profile of unique molecular identifiers (UMIs) and UMIs/gene, we removed cells with less than 600 UMIs and less than 1.2 UMIs/expressed gene. The remaining 14,121 cells had a mean of 5275 UMI counts/cell and 1.9 counts/expressed gene (Fig. 1A; Fig. S3). Based on recent scRNA-seq analysis of the entire adult mouse nervous system (Zeisel et al., 2018), we identified non-neural cells, for example, vascular cells, blood cells and microglia, and because these would not have been targeted by *Sox1-Cre* (Yaghmaeian Salmani et al., 2018), we excluded them from further analysis (Fig. 1A). This yielded 13,579 hypothalamic neural cells, denoted ‘All-Hypo’; 6953 cells from control and 6626 cells from *Eed-cKO* embryos (Fig. 1B,C), visualised by Uniform Manifold Approximation and Projection (UMAP) embedding, based upon the top-300 most variable genes (see Materials and Methods; Table S1).

Recent studies have highlighted the strength of pseudobulk approaches for assessing differential gene expression (DE) in scRNA-seq data (Squair et al., 2021). To this end, we applied a previously developed limma empirical Bayes analysis pipeline (Law et al., 2014). Analysing the All-Hypo cells using this approach revealed 2549 differentially expressed genes (DEGs), when comparing *Eed-cKO* with control (Table S2); 1679 genes were upregulated and 870 were downregulated (Fig. 1D). Although *Eed* was downregulated, *Ezh1*, *Ezh2* and *Suz12* were not affected (Table S2).

To gain insight into the biological processes, molecular function and the epigenetic modifications of the genes affected in *Eed-cKO* we performed gene set enrichment analysis (GSEA) against the GO biological process, ‘GO_Molecular_Function_2018’ and the ‘ENCODE_Histone_Modifications_2015’ databases (Ashburner et al., 2000; Davis et al., 2018; Gene Ontology Consortium, 2021). This analysis revealed an overall upregulation of genes involved in developmental patterning and a downregulation in genes involved in synapse formation (Fig. S4A,B; Table S3). Regarding the epigenomic signature of affected genes, both up- and downregulated genes showed enrichment for the bivalent state, i.e. with both H3K27me3 and H3K4me1/3 marks in ENCODE (Fig. S4C; Table S3). Downregulated genes also displayed enrichment for the H3K27me3 mark (Fig. S4C; Table S3).

### Single cell transcriptional profiling reveals cell cycle inhibitor upregulation and brain posteriorization in *Eed* mutants

Our previous study of *Eed-cKO* mutants revealed reduced proliferation in the developing telencephalon, accompanied by upregulation of two members of the Cip/Kip family of cell cycle inhibitors; *Cdkn1a* and *Cdkn1c* (Yaghmaeian Salmani et al., 2018). In line with these findings, analysing the All-Hypo cells using UMAP embedding and DE analysis revealed upregulation of *Cdkn1c*, as well as of three of the four members of the INK4 family of cell cycle inhibitors, *Cdkn2a*, *Cdkn2b* and *Cdkn2c* (Fig. 1D; Table S2).

Turning to developmental regulatory genes, UMAP embedding and DE analysis revealed that 23 out of the 39 Hox homeotic genes were upregulated in the *Eed-cKO* mutants (Fig. 1E-G; Table S2). This is in line with our previous analysis of the telencephalon, using bulk RNA-seq, which showed upregulation of all 39 Hox genes to levels matching their expression in the spinal cord (Yaghmaeian Salmani et al., 2018). Hence, the scRNA-seq and immunostaining analysis revealed that *Eed-cKO* mutants displayed posteriorization and a reduced proliferative gene expression profile in the developing hypothalamus.

### Single cell transcriptional profiling identified all major hypothalamic cell types at E18.5

**Fig. 2.**
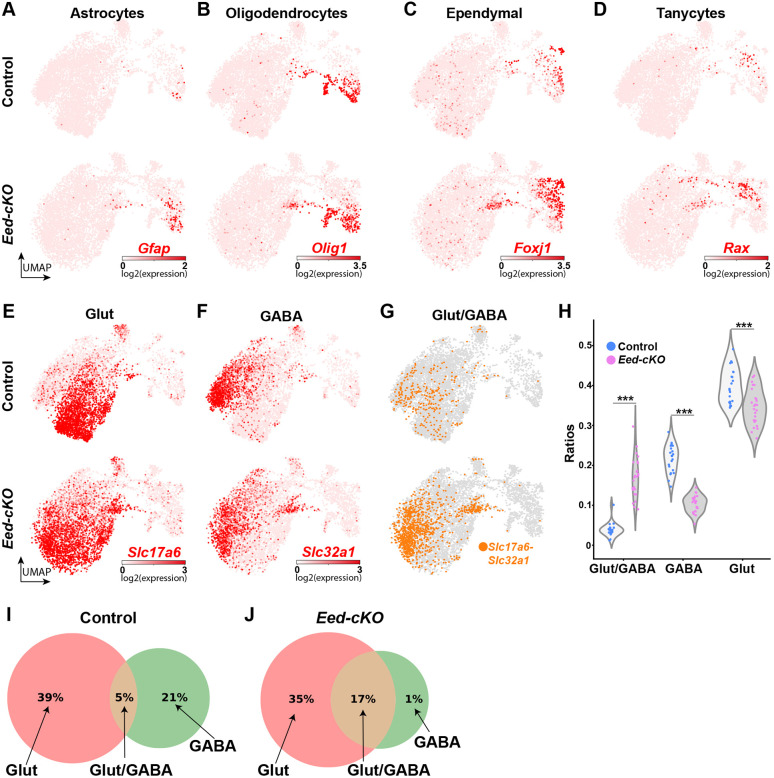
***Eed-cKO* mutants display more Glut/GABA cells.** (A-F) UMAP embedding, based upon 300 DEGs (Table S1), of E18.5 All-Hypo cells, showing astrocytes (*Gfap*; A), oligodendrocyte precursors (*Olig1*; B), ependymal cells (*Foxj1*; C), tanycytes (*Rax*; D), glutamatergic neurons (*Slc17a6*; E) and GABAergic neurons (*Slc32a1*; F). (G) Glut/GABA co-expression revealed by *Slc32a1* and *Slc17a6* expression. (H) Violin plots of the ratios of Glut, Glut/GABA and GABA cells detected per embryo stratified by control and *Eed-cKO* reveal an increase in Glut/GABA cells and a decrease in GABA and Glut cells in *Eed-cKO* (***adjusted *P*-value<1e-3; *t*-test on centred-log-ratios, see Table S10 for details). (I,J) Venn diagrams summarizing the percentage of Glut, Gluta/GABA and GABA cells in control (I) and *Eed-cKO* (J).

The ectopic expression of many posteriorly expressed TFs suggested that hypothalamic cell specification may be strongly affected in the *Eed-cKO* mutants. To address this issue, we first analyzed the generation of broader hypothalamic cell types (Chen et al., 2017; Kim et al., 2020; Romanov et al., 2020, 2017). UMAP embedding of All-Hypo cells revealed that the major cell types were generated in *Eed-cKO* mutants, including GABAergic neurons (*Slc32a1*), glutamatergic neurons (*Slc17a6*), oligodendrocytes (*Olig1*), astrocytes (*Gfap*), ependymal cell (*Foxj1*) and tanycytes (*Rax*) (Fig. 2A-F). However, there was an increase in *Gfap*-, *Foxj1*- and *Rax*-expressing cells, evident both in the UMAPs and DE analysis (Fig. 2B-D; Table S2), and well as in Gfap immunostaining (Fig. S5A,B).

We also observed a striking increase in the overlap between glutamatergic and GABAergic markers (*Slc32a1* and *Slc17a6*) (Fig. 2E-G). Although Glut/GABA cells are rare in the cortex (Zeisel et al., 2015), previous adult scRNA-seq analysis found these cells to be more common in the hypothalamus (Romanov et al., 2017). Quantification of Glut/GABA double-positive cells confirmed the presence of these cells in control and confirmed a significant increase in the proportion of these cells in the *Eed-cKO* mutants (Fig. 2H-J).

To address the identity of the extra Glut/GABA cells, we probed their gene expression profile. In control, Glut/GABA cells show differential expression of 136 genes when compared with Glut cells, and 222 genes when compared with GABA cells (Fig. S6A,B; Table S4). In *Eed-cKO* mutants, Glut/GABA cells also display differential expression of 57 and 92 of the Glut/GABA-specific genes when compared with Glut and GABA cells, respectively. However, *Eed-cKO* mutants also display upregulation of many additional genes in Glut/GABA cells, indicating that these cells may not fully mimic wild-type Glut/GABA cells (Fig. S6A-C; Table S4).

**Fig. 3.**
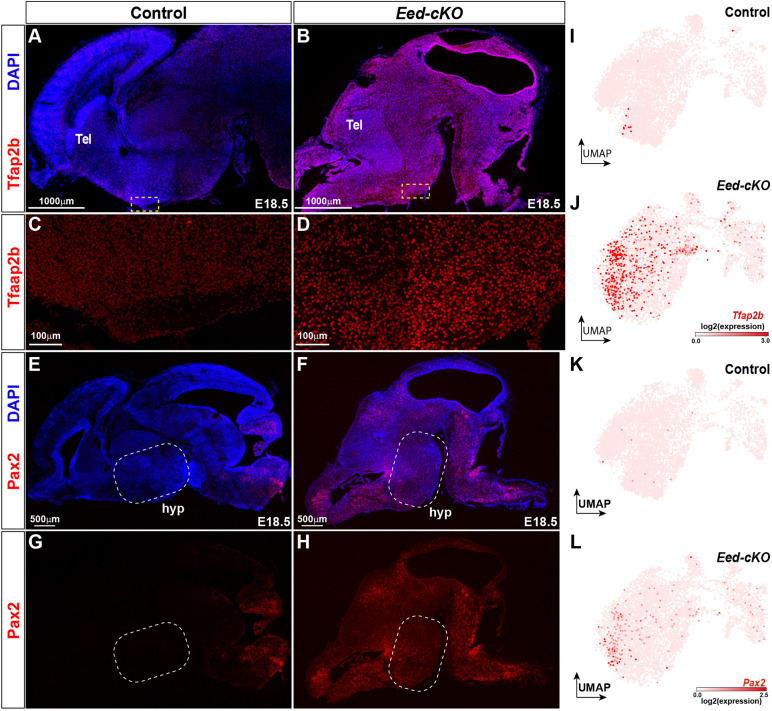
***Eed-cKO* mutants display ectopic Tfap2b and Pax2 expression in the hypothalamus.** (A-H) Staining for DAPI, Tfap2b (A-D) and Pax2 (E-H) in sagittal sections of E18.5 brains in control and *Eed-cKO*. Dashed squares in A,B,E,F delineate regions of hypothalamic tissue magnified in C,D,G,H. In control, Tfap2b and Pax2 expression is observed in the mid- and hindbrain regions. In *Eed-cKO* mutants, Tfap2b and Pax2 expression expands into the anterior brain, including the hypothalamus. (I-L) UMAP embedding of E18.5 hypothalamic scRNA-seq All-Hypo cells revealing the ectopic expression of *Tfap2b* (I,J) and *Pax2* (K,L) in control and *Eed-cKO*.

The increased proportion of Glut/GABA co-expressing cells was accompanied by a reduction in the proportion of both GABA and Glut cells (Fig. 2H-J). This effect prompted us to scrutinize the TF expression changes in the *Eed-cKO*. We noted that *Tfap2a* and *Tfap2b*, known determinants of cerebellar GABAergic cell fate (Zainolabidin et al., 2017), as well as *Pax2*, *Pax8* and *Lhx5*, known determinants of spinal cord GABAergic cell fate (Cheng et al., 2004; Pillai et al., 2007), were among the genes ectopically expressed. This was evident from UMAP embedding and DE analysis, as well as from Tfap2a and Pax2 immunostaining (Fig. 1G, Fig. 3A-L; Table S2).

Hence, although all major hypothalamic subtypes were generated in *Eed-cKO*, there was an increase in several non-neuronal cell types. In addition, there was an increase in the proportion of Glut/GABA cells, accompanied by the ectopic expression of the *Tfap2a*, *Tfap2b*, *Pax2*, *Pax8* and *Lhx5* GABAergic determinants.

### Single cell transcriptional profiling reveals limited effects on hypothalamic cell fate specification in *Eed* mutants

**Fig. 4.**
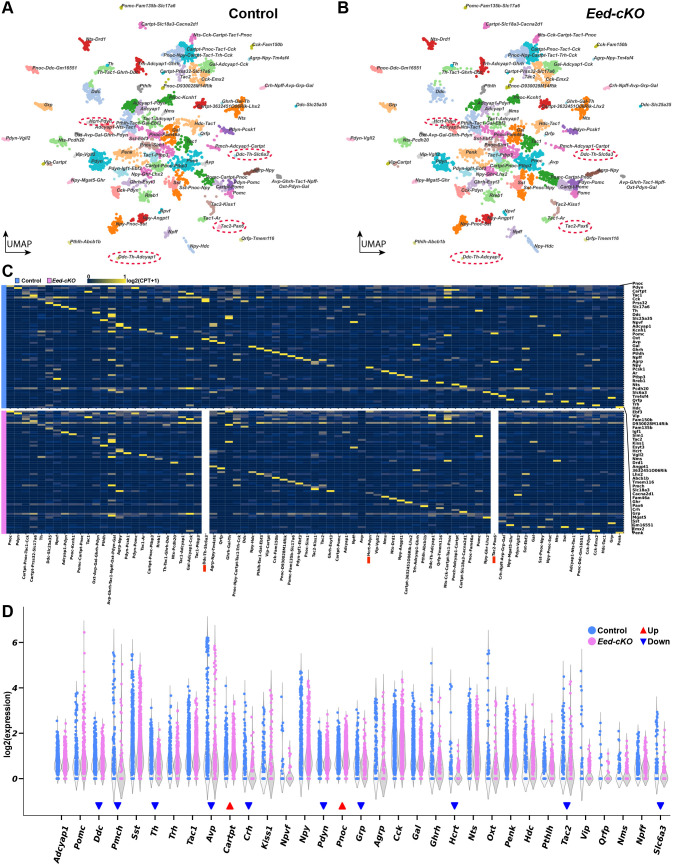
***Eed-cKO* is not necessary for most NP-DA cell types.** (A,B) UMAP embedding of hypothalamic NP-DA cells, based on 122 genes (Table S1), in control (A) and *Eed-cKO* (B) at E18.5. Each cluster is labelled by the combination of NP-DA marker genes upregulated in that cluster. (C) Heatmap displaying average gene expression of the NP-DA marker genes in each UMAP cell cluster, in control (blue, top) and *Eed-cKO* (pink, bottom). Gene expression is measured in log2(CPT+1), scaled between 0 and 1 along each row (see Materials and Methods). The *Ddc-Th-Slc6a3*, *Tac2-Pax6* and *Hcrt* clusters are absent in *Eed-cKO,* and hence a column of 0 expression was depicted. (D) Expression levels of NP-DA marker genes in control and *Eed-cKO* at E18.5 (see Table S2 for analysis).

Next, we focused on the generation of more discrete neuronal subtypes. We analyzed the All-Hypo cells for their expression of 29 neuropeptide (NP) genes (Table S1), the *Th* and *Ddc* dopamine markers (DA), and the *Hdc* histaminergic marker, and thereby identified 9590 ‘NP-DA’ cells within the All-hypo cells; 4799 from control and 4791 from *Eed-cKO* embryos (Table S5). We created UMAP embedding of the NP-DA cells using the NP-DA genes and 93 additional genes found to be most correlated with the expression of the NP-DA marker genes (Table S1). In control, clustering and UMAP visualisation based upon these marker genes identified 79 major cell clusters (Fig. 4A,C; Table S6). Several clusters displayed co-expression of neuropeptides, i.e. *Agrp-Npy*, *Avp-Gal*, *Kiss1-Tac2* and *Npy-Sst*, which is in agreement with previous scRNA-seq expression analysis in the adult and/or embryonic hypothalamus (Chen et al., 2017; Romanov et al., 2020, 2017; Zhang et al., 2021b). Expression of *Th* and *Ddc* is broader than DA neurons (Björklund and Dunnett, 2007), and *Th*-*Ddc* co-expression is only detected in two smaller cell clusters, which also express *Slc6a3*, identifying them as bona fide DA neurons (Fig. 4A,C). Two neuropeptide genes (*Galp*, *Prlh*) were not detected. We analyzed the potential sexual dimorphism of the 79 major NP-DA clusters but found no evidence for significant dimorphism (Table S5). Similar to previous studies (Chen et al., 2017; Romanov et al., 2017), we found that in the control, the vast majority of neuropeptide neurons co-express either Glut or GABA markers, whereas Glut/GABA primarily co-expressed with *Npff* (Fig. S7A).

Turning to the *Eed-cKO* mutants, despite the reduced proliferation and the ectopic expression of many posterior TFs, the UMAP embedding revealed surprisingly limited effects upon NP-DA cell specification in the E18.5 hypothalamus, as evidenced by the generation of the majority of the 79 NP-DA subtypes (Fig. 4). This specification of NP-DA subtypes occurred despite the ectopic expression of Hox and other regulatory genes within most cell clusters (Fig. S8A,B). The increase in Glut/GABA marker co-expression in *Eed-cKO* occurred in several different neuropeptide cells (Fig. S7B).

### Single cell transcriptional profiling reveals loss of DA neurons in *Eed* mutants

**Fig. 5.**
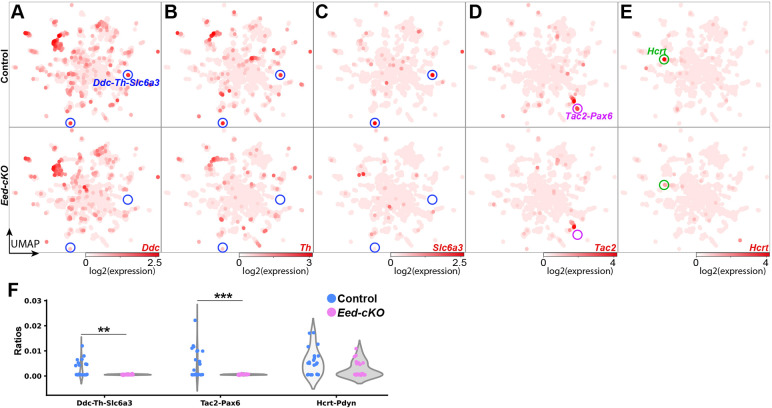
***Eed-cKO* is necessary for DA, *Hcrt* and *Tac2* cells.** (A-E) UMAP embedding of E18.5 NP-DA cells, based upon 122 DE genes (Table S1), showing expression of *Ddc* (A), *Th* (B), *Slc6a3* (C), *Tac2* (D) and *Hcrt* (E). Two prominent clusters co-express *Ddc*-*Th*-*Slc6a3*, and these cluster are largely missing in *Eed-cKO* (A-C). Similarly, one specific *Tac2* cluster and the *Hcrt* cluster are largely absent in *Eed-cKO* mutants (D,E). (F) Violin plots of the ratios of *Ddc*-*Th*-*Slc6a3*, *Tac2*-*Pax6* and *Hcrt*-*Pdyn* cell types in control and *Eed-cKO* embryos (see Table S2 for analysis). **adjusted *P*-value<0.01, ***adjusted *P*-value<0.001; *t*-test on centred-log-ratios (see Table S5 for details).

Although the scRNA-seq and immunostaining analysis revealed that most NP-DA cell subtypes were generated in *Eed-cKO*, we did note several specific effects. Interestingly, UMAP embedding revealed that one of the *Ddc-Th-Slc6a3* clusters was largely lost in *Eed-cKO* mutants, and the other was reduced in size (Fig. 4A,B). In line with this finding, *Ddc*, *Th* and *Slc6a3* were all significantly downregulated in *Eed-cKO* mutants (Fig. 4C,D; Table S2; Fig. 5A-C,F). Studies of hypothalamic DA cells have revealed the involvement of several TFs, including Dlx1, Otp, Sim1, Satb2 and Arnt2 (Biran et al., 2015; Orquera et al., 2016; Yee et al., 2009; Zhang et al., 2021a). Because the *Ddc-Th-Slc6a3* cells are largely missing in *Eed-cKO*, we could not determine the expression of these genes in the DA cells. However, DE analysis of the All-Hypo cells revealed that *Dlx1* and *Arnt2* were both downregulated in All-Hypo cells (Table S2). For unclear reasons, *Otp* was neither detected in control nor *Eed-cKO* (Table S2). Previous studies identified *Onecut3* as a specific marker for the A14 DA group (Romanov et al., 2017), but we did not observe any changes in *Onecut3* expression (Table S2).

### Loss of *Hcrt* and *Tac2*-*Pax6* neurons in *Eed* mutants

Turning to neuropeptide cells, the UMAP embedding of NP-DA cells indicated that most of the neuropeptide-expressing cell clusters were still present in *Eed-cKO* (Fig. 4A,B). However, DE analysis revealed significant downregulation of seven genes and upregulation of two genes (Fig. 4C,D; Table S2). The DE effects on these nine neuropeptide genes were apparent also in the individual gene expression UMAPs and manifested as either an overall change in expression (*Pmch*, *Cartpt*, *Pdyn*, *Pnoc*), the loss of expression in one or several clusters (*Avp*, *Crh*, *Grp*, *Tac2*) or the loss of expression in the one single cluster (*Hcrt*) (Fig. 5D,E; Figs S9 and S10; Table S2). For *Tac2*, the affected cluster selectively co-expressed *Pax6* (Figs 4C and 5D-F; Table S6). Because the *Tac2-Pax6* cells are lost in *Eed-cKO*, we could not determine the expression of *Pax6* or other genes in the mutant *Tac2-Pax6* cells (Table S6), but *Pax6* was not affected by DE analysis (Table S2). There were also changes apparent in the expression of several other neuropeptide genes based upon the individual UMAPs. However, the scarcity of cells, overall or in specific clusters, resulted in these not being flagged as significantly affected by DE analysis (e.g. *Agrp*, *Ghrh*, *Kiss1*, *Nms*, *Npff*, *Qrfp*, *Vip*) (Figs S9 and S10; Tables S2 and S6).

Next, we probed the effects upon some of the strongly affected neuropeptide genes by immunostaining. The UMAP analysis revealed several *Avp*-expressing clusters, one of which was absent in *Eed-cKO* mutants (Fig. S11H,I). Immunostaining and *in situ* hybridization of control at E18.5 revealed two cell groups, one dorsal and one ventral (Fig. S11A-D). In *Eed-cKO* mutants, as anticipated from the UMAP analysis, the generation of Avp neurons was still evident. However, the ventral group was broken up into several subgroups (Fig. S11E-G).

**Fig. 6.**
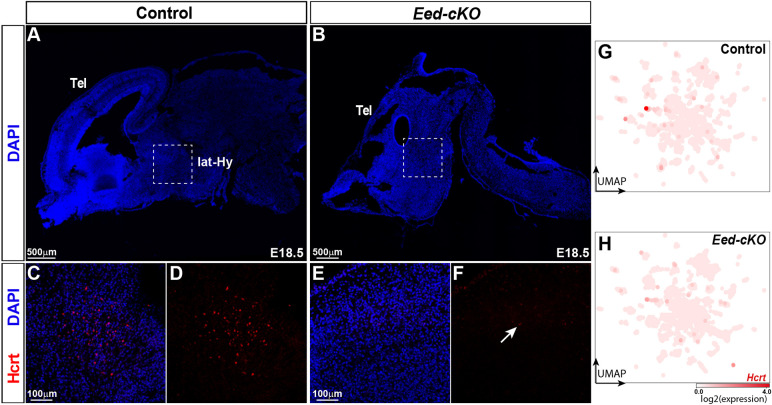
***Eed* is crucial for Hcrt neurons.** (A,B) Staining for DAPI in sagittal sections of E18.5 control and *Eed-cKO* mutant brains. Dashed boxed areas delineate the lateral hypothalamus (lat-Hy). (C-F) Magnifications of boxed areas in A and B showing staining for DAPI and Hcrt in the lateral hypothalamus in control (C,D) and *Eed-cKO* (E,F) embryos. A cluster of Hcrt cells is present in the control, whereas *Eed-cKO* mutants only show occasional weakly expressing cells. (G,H) UMAP embedding of E18.5 hypothalamic scRNA-seq NP-DA cells, with each cell coloured according to the expression level of *Hcrt* in control and *Eed-cKO*.

*Eed-cKO* mutants displayed a near complete loss of cells highly expressing *Hcrt* in the UMAP and reduced expression levels (Figs 4A-D, 5E,F, 6G,H; Tables S2 and S6). This observation was confirmed by immunostaining (Fig. 6A-F). Previous studies have identified several TFs that are involved in Hcrt cell specification, including Dbx1, Ebf2 and Lhx9 (Dalal et al., 2013; De La Herran-Arita et al., 2011; Liu et al., 2015; Sokolowski et al., 2015), and in control *Hcrt* cells, *Lhx9* is differentially expressed in *Hcrt* cells when compared with other NP-DA clusters (Table S6). Because the *Hcrt* cells are largely lost in *Eed-cKO*, we could not determine the expression of *Dbx1*, *Ebf2* or *Lhx9* in Hcrt cells in the mutant (Table S5). However, DE analysis of All-Hypo cells revealed that *Ebf2* was downregulated in *Eed-cKO* (Table S2). Despite the gradual loss of H3K27me3, we did not observe any apparent correlation between the specific NP-DA cells affected and the onset of their marker genes (Table S7).

### scRNA-seq at earlier stages reveals reduction of *Lhx9*-expressing progenitor cells in *Eed* mutants

To better understand the origins of the main phenotypes observed at E18.5 we conducted scRNA-seq analysis of control and *Eed-cKO* at earlier stages; E13.5 and E15.5. We dissected the hypothalamus from 18 control (*Eed^fl/fl^*) and 19 *Eed-cKO* embryos (eight female and ten male embryos for control; ten female and nine male embryos for *Eed-cKO*). Cells were dissociated and 120 samples were sequenced on the Illumina/Bio-Rad ddSEQ platform (22 female and 27 male samples for control; 36 female and 35 male samples for *Eed-cKO*), which yielded a total of 51,540 high-quality cells. Following the same biological filtering process as for E18.5, we yielded a total of 50,469 hypothalamic neural cells (All-Hypo); 20,959 cells from control and 29,510 cells from *Eed-cKO* embryos (Fig. S12A-D).

**Fig. 7.**
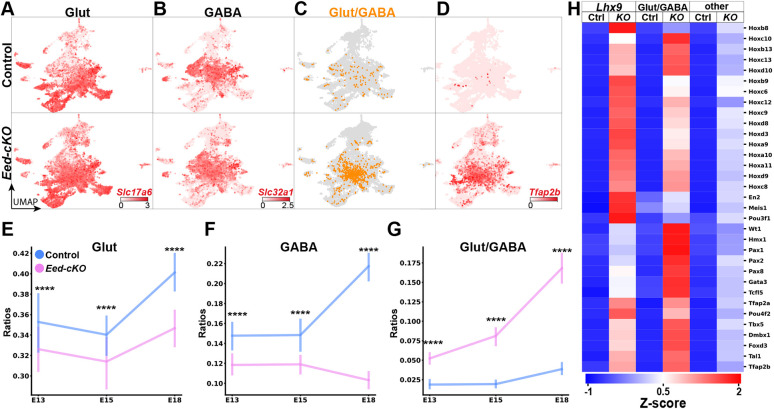
**Developmental onset of Glut/GABA co-expression.** (A-D) UMAP embedding of hypothalamic cells from E13.5, E15.5 and E18.5, depicting expression of *Slc17a6* (A), *Slc32a1* (B) and *Tfap2b* (D). Glut/GABA co-expression (C) and *Tfap2b* expression is limited in control but increases in *Eed-cKO* mutants. (E-G) Quantification of Glut-, GABA- and Glut/GABA-expressing cells at developmental time points (see Tables S9 and S10 for analysis). In *Eed-cKO*, Glut/GABA cells increase at the expense of both Glut and GABA cells. (H) Expression analysis of *Lhx9* cells, Glut/GABA cells and ‘other’ cells, at E13.5-E18.5. In *Eed-cKO*, *Lhx9* and Glut/GABA cells show ectopic expression of many developmental TFs, and display upregulation to a greater extent than all ‘other’ cells.

We first addressed the development of Glut, GABA and Glut/GABA cells. Similar to what was observed at E18.5, we noted significantly higher numbers of Glut/GABA cells in *Eed-cKO* mutants at E13.5 and E15.5 (Fig. 7A-C,E-G; Table S9). Filtering out potential doublet cells still yielded the significant increase in Glut/GABA cells at E13.5, E15.5 and E18.5 (Fig. S13; Table S10). Again, gene expression analysis revealed ectopic expression of the GABA neuron determinants *Tfap2a/b*, *Pax2*, *Pax8* and *Lhx5* (Fig. 7D,H; Fig. S12E; Table S2).

As outlined above, we noted an increase in tanycytes (*Rax*) and ependymal cells (*Foxj1*) at E18.5. In an attempt to delineate the origin of these effects, we leaned against recent developmental trajectory analysis of tanycytes and ependymal cells (Kim et al., 2020). However, although we observed an increase in both cell types at all three developmental stages in *Eed-cKO*, analysis of their developmental trajectory and gene expression profiles did not reveal any apparent aberrancies when examining the expression of previously identified lineage genes (Fig. S14A-D).

With regards to DE including the greater number of samples and earlier time points, we observed that almost all of both up- and downregulated genes were previously identified from the E18 DE analysis (Fig. S4E,F). Furthermore, testing for a time-specific effect of *Eed-cKO* identified very few DE genes (total 35), indicating very weak temporal changes in the effect of *Eed-cKO* across the time points sampled (Fig. S4G).

With the increased number of samples, we also decided to probe for sex differences in both control and *Eed-cKO*. Sexual dimorphism was evident in the control by DE of 13 genes (nine upregulated in females; four upregulated in males), including some well-known dimorphic genes such as *Xist* (up in females) and *Kdm5d* (up in males) (Fig. S4H; Table S2). In *Eed-cKO*, no sex-specific effect of the mutant was observed, indicating the same DE in the control between sexes was still present (Fig. S4I; Table S2).

Next, we focused on the three restricted neuronal cell types affected at E18.5. Regarding, the DA cells (*Th*-*Ddc*-*Slc6a3*), in control we observed one main cluster of *Th*-*Ddc*-*Slc6a3*-expressing cells (Fig. S15A). In *Eed-cKO* mutants, this cluster was present at E13.5 and E15.5, but significantly reduced at E18.5 (Fig. S15A-C). Hence, in *Eed-cKO* mutants DA neurons appear to initially be generated and/or specified but lost at later stages.

For *Tac2*, control embryos displayed several clusters of cells, one of which was absent in *Eed-cKO* across all stages (Fig. S16E,F). This cluster also expressed *Pax6*, which appeared to be downregulated in this branch in mutants (Fig. S16E,F). These findings indicate that *Tac2-Pax6* cells are never generated and/or specified in the mutants.

For *Hcrt*, a single cluster was evident in control also at the earlier embryonic stages, and this cluster was absent in *Eed-cKO* mutants (Fig. S16A). This indicates that *Hcrt* cells are never generated and/or specified in the mutants. Because Lhx9 is one of the few known TFs involved in *Hcrt* cell specification, we examined *Lhx9* expression in the temporal UMAP space. This revealed a specific Hes5−, Sox2+, Lhx9+ progenitor population that displayed loss/reduction in cell numbers *Eed-cKO* (Fig. S16B-D). Gene expression analysis of the *Lhx9* group of cells between control and *Eed-cKO*, as well as against all mutant cells (Table S11), revealed significant upregulation of many developmental regulators in the *Lhx9* population, including 29/39 of the Hox transcription factors (Fig. 7H; Table S11). We observed significantly greater ectopic expression of developmental regulators in both the *Lhx9* and Glut/GABA cells compared with all other cells (Fig. 7H; Table S11), revealing that these two cell populations were particularly affected.

Therefore, the major phenotypes observed in *Eed-cKO* at E18.5, the increase in the proportion of Glut/GABA co-expressing cells, as well as the loss/reduction of *Hcrt* and *Tac2-Pax6* cells were also apparent at earlier stages. In contrast, the loss/reduction of DA neurons appears to play out at later embryonic stages.

## DISCUSSION

### Gradual loss of H3K27me3 in *Eed* mutants

Epigenomic methylation marks are generally viewed as stable over time. However, they are subjected to two main removal cues: demethylation and replication-mediated dilution. How do these removal dynamics fit with the observed gradual reduction of H3K27me3 in *Eed-cKO*?

Demethylation of H3K27me3 is chiefly mediated by *Kdm6a* (*Utx*) and *Kdm6b* (*JMJD3*) (Arcipowski et al., 2016). However, although mutation of *Kdm6a* and/or *Kdm6b*, in mouse or embryonic stem cells, did result in elevated H3K27me3 levels at specific loci, there was no global increase in H3K27me3 levels (Burgold et al., 2008; Park et al., 2014; Tang et al., 2020; Wijayatunge et al., 2018). Similarly, in *Drosophila*, *Utx* mutation did not alter the dynamics of H3K27me3 reduction at a specific target locus in the developing wing disc (Laprell et al., 2017). Therefore, although demethylation mediated by Kdm6a/b modulates H3K27me3 levels, it does not appear to play an instrumental role in counteracting PRC2 activity at all target loci.

By contrast, DNA replication can efficiently reduce the levels of the H3K27me3 mark by H3 dilution, as new nucleosomes form post replication. Studies in *Drosophila* have found that removal of a Polycomb Response Element (PRE) at a target locus resulted in the gradual reduction of H3K27me3, with some 50% reduction per cell cycle, and loss of transcriptional repression after only one cell cycle (Coleman and Struhl, 2017; Laprell et al., 2017). Similarly, studies of the mouse intestine have revealed that loss of PRC2 activity (by *Eed* or *Ezh2* mutation) resulted in the gradual loss of H3K27me3 levels, also with an estimated 50% reduction per cell cycle (Jadhav et al., 2020).

To circumvent early embryonic lethality we inactivated *Eed* using the early neuroectodermal Cre deleter, *Sox1-Cre* (active at E8.5) (Takashima et al., 2007). This resulted in a gradual loss of the H3K27me3 mark in the *Eed-cKO* mutants, with reduction in immunostaining at E10.5 and complete loss at E11.5, in the telencephalon, spinal cord and hypothalamus (Yaghmaeian Salmani et al., 2018). We envision that some of the delay in H3K27me3 reduction results from the delay in deletion of both gene copies, as well as the degradation of wild-type *Eed* mRNA and Eed protein, the latter of which may be especially slow for Eed protein that is associated with the PRC2 complex. In addition, a major part of the gradual reduction of H3K27me3 levels is likely connected to proliferation dynamics. Progenitor cycling speeds have not been mapped in the early mouse hypothalamus, but studies of the E11 mouse telencephalon has revealed neuroepithelial progenitor cell cycle speeds of 8-10 h (Caviness et al., 1995; Takahashi et al., 1995). Considering these studies, the gradual loss of the H3K27me3 mark in *Eed-cKO* mutants, being reduced at E10.5 and lost at E11.5, i.e. 2-3 days and hence some six to nine cell divisions after *Eed* inactivation at E8.5, is consistent with a replication-mediated dilution of modified H3 histones.

Logically, our GO analysis of *Eed-cKO* mutants revealed an enrichment the H3K27me3 mark on differentially expressed genes. However, there was also an enrichment of the activating mark H3K4me1/3. This is in line with the notion that developmental genes, in particular TF genes, often display both of these marks, the so called bivalent status (Blanco et al., 2020; Sun et al., 2021).

### Reduced proliferation in *Eed-cKO* mutants

Previous studies of *Eed-cKO* mutants revealed reduced proliferation in the telencephalon (Telley et al., 2019; Yaghmaeian Salmani et al., 2018), which was accompanied by increased expression of the Cip/Kip cell cycle inhibitors *Cdkn1a* and *Cdkn1c* (Yaghmaeian Salmani et al., 2018). Here, we observed reduced proliferation also in the hypothalamus, upregulation of *Cdkn1c*, as well as of three of the four members of the INK4 family of cell cycle inhibitors, *Cdkn2a*, *Cdkn2b* and *Cdkn2c*, in the E18.5 scRNA-seq data. It is likely that the upregulation of many of the Cip/Kip and INK4 cell cycle inhibitor genes is a major reason for the reduced proliferation in *Eed-cKO* mutants.

The reduced proliferation in the hypothalamus begs the question of which cells are not being generated. Recent scRNA-seq analysis of the developing hypothalamus has revealed that neurons are generally born earlier than other cell types, appearing at E10 onward, and that glia, ependymal cells and tanycytes appear from E16 onward (Kim et al., 2020; Romanov et al., 2020; Zhang et al., 2021b). Our UMAP embedding of the All-Hypo cells did not reveal striking loss of any major cell type, i.e. GABAergic neurons, glutamatergic neurons, astrocytes, oligodendrocytes, ependymal cells and tanycytes. However, we observed an increase in Glut/GABA co-expressing cells. Single expressing Glut and GABA cells, as well as Glut/GABA co-expressing cells, are born early during hypothalamic development, but are also added throughout embryogenesis (Kim et al., 2020; Romanov et al., 2020; Zhang et al., 2021b). Against this backdrop, it is not apparent whether/how the reduced proliferation observed in *Eed-cKO* mutants could act to increase the number of Glut/GABA co-expressing cells.

Regarding NP-DA subtypes, although the actual birthdate for most hypothalamic neuronal subtypes has not been determined, the onset of NP-DA marker expression has been analyzed in most cases (Table S7). Assuming a gradual loss of the H3K27me3 mark, a logical effect would be the loss of late-expressing NP-DA markers, indicating a failure to generate late-born neurons. However, the subtypes affected do not apparently link to marker gene expression onset, with genes activated both early (E11.5) and late (E15.5) being affected or, alternatively, unaffected (Table S7). Hence, the differential effects upon discrete cell subtypes are not readily explained by differential birth dates.

Previous analysis in the telencephalon revealed that *Eed-cKO* mutation primarily affected the proliferation of daughter cells, as opposed to progenitor cells (Yaghmaeian Salmani et al., 2018). Recent studies, including genetic lineage tracing, have revealed a complex repertoire of progenitor and proliferative daughter cells in both the mouse and human hypothalamus, which in many aspects are similar to the developing telencephalon (Zhang et al., 2021b; Zhou et al., 2020). It is tempting to speculate that the selective effects of *Eed-cKO* upon cellular subtypes born both early and late may relate to the lineage topology of different hypothalamic lineages, an issue of which we still know very little.

### *Eed-cKO* triggers posteriorization of the brain

A well-conserved role of PRC2 in bilaterians is to confine Hox homeotic gene expression to the posterior CNS (Isono et al., 2005; Li et al., 2011; Struhl, 1983; Struhl and Akam, 1985; Suzuki et al., 2002; Wang et al., 2002; Yaghmaeian Salmani et al., 2018). Our previous studies of *Eed-cKO* mutants confirmed this role for PRC2, and we observed ectopic expression of all 39 Hox homeotic genes in the E15.5 mouse forebrain, as revealed by bulk RNA-seq (Yaghmaeian Salmani et al., 2018). We also observed several other posteriorly expressed TFs being ectopically expressed in the forebrain. Here, using scRNA-seq, we found similar effects in the developing hypothalamus. A growing body of work is pointing to the role of Hox genes in regulating cell cycle genes and repressing proliferation (Economides et al., 2003; Monedero Cobeta et al., 2017; Yaghmaeian Salmani et al., 2018). Strikingly, in *Drosophila*, simultaneous removal of Hox genes can rescue reduced proliferation in *esc* mutants (the *Drosophila* orthologue of *Eed*) (Bahrampour et al., 2019). These studies indicate that the reduced hypothalamic proliferation observed in *Eed-cKO* may be a direct result of the posteriorization of the entire fore- and midbrain, and the ectopic expression of posterior Hox homeotic genes.

### Selective involvement of *Eed* upon hypothalamic cell fates

In *Eed-cKO* mutants, despite the reduced proliferation and upregulation of many posteriorly expressed developmental regulators, we observed that most neural subtypes were still generated. One possible explanation for the limited effects observed may be that *Eed-cKO* results in the loss of H3K27me3 after the time that many important developmental decisions, i.e. patterning of the hypothalamic progenitor region, have already played out between E8 and E11 (Alvarez-Bolado, 2019; Bedont et al., 2015; Blackshaw et al., 2010; Burbridge et al., 2016; Ferran et al., 2015; Puelles and Rubenstein, 2015; Xie and Dorsky, 2017). Nevertheless, major aspects of neurogenesis and cell specification do occur after E11.5, and although there is extensive mis-regulation of many genes it is surprising that H3K27me3 is apparently not crucial for many of the genetic cascades that govern cell fate.

Although many aspects of hypothalamic development appeared to be unperturbed, we did observe an increase the proportion of Glut/GABA co-expressing cells, apparent at all stages analyzed. Interestingly, we observed upregulation/ectopic expression of the cerebellar/spinal cord GABA cell fate determinants *Tfap2a*, *Tfap2b*, *Pax2*, *Pax8* and *Lhx5* (Cheng et al., 2004; Pillai et al., 2007; Zainolabidin et al., 2017), and their expression showed extensive overlap with Glut/GABA co-expressing cells. This raises the possibility that upregulation/ectopic expression of these GABA determinants may underlie the increase in Glut/GABA co-expressing cells, although it is unclear why these changes in TF expression would not extinguish the Glut fate.

In addition, *Eed-cKO* mutants also displayed the reduction/loss of DA, Hcrt and Tac2-Pax6 cells. Although the absence of these cells in *Eed-cKO* mutants hindered detailed analysis of the underpinnings of these phenotypes, we observed that two of the three known regulators of hypothalamic DA cells, *Dlx1* and *Arnt2*, were downregulated. Similarly, we observed downregulation of one of the three known regulators of *Hcrt* cells, *Ebf2*. Downregulation of these TFs may explain the absence of DA and Hcrt cells. Indeed, analysis of earlier stages revealed that a subset of *Lhx9*-positive progenitors (Sox2+, Hes5−) are missing in the mutant. Specification of the different *Tac2* neuronal subtypes has not been previously addressed, and hence the underpinning of this phenotype is currently unclear.

Are there any commonalities between DA, Hcrt and Tac2-Pax6 neurons that could explain the absence of these three cell types? The specific progenitor region of origin for the different hypothalamic DA neuron subtypes (A11, A12, A14, A15), Hcrt or Tac2-Pax6 neurons is not clear, but their final cell body locations (DA neurons medial, Hcrt neurons lateral and Tac2 neurons in several regions) argue against a common origin. Similarly, the non-overlapping sets of upstream regulators, Dlx1, Otp, Sim1, Satb2 and Arnt2 for DA neurons (Biran et al., 2015; Orquera et al., 2016; Yee et al., 2009; Zhang et al., 2021a), and Dbx1, Ebf2 and Lhx9 for Hcrt neurons (Dalal et al., 2013; De La Herran-Arita et al., 2011; Liu et al., 2015; Sokolowski et al., 2015), also argue against a common origin, as well as against a common genetic pathway. Interestingly, knockout of the H3K4-methyltransferase *Mll4* (*Kmt2d*) gene in the developing hypothalamic arcuate nucleus also resulted in highly selective effects on neuronal subtype specification (Huisman et al., 2021).

### Implications for sensitivity to aberrant PRC2 activity in humans

A growing body of work points to the role of epigenetics in gating entry into puberty, in particular with respect to controlling the expression of neuropeptide genes involved in triggering puberty, such as *Kiss1* and *Tac2* (*Tac3*) (Aylwin et al., 2019; Shalev and Melamed, 2020). This pertains specifically to the PRC2 complex, which plays a major role in gating the elevated expression of the *Kiss1* and *Tac2* genes necessary for puberty. Analysis of several PRC2 genes revealed that *Eed* gene expression is downregulated in the hypothalamus pre-puberty, followed by *Kiss1* and *Tac2* upregulation. Strikingly, overexpression of *Eed* resulted in repression of *Kiss1* gene expression and an inhibition/delay of puberty (Lomniczi et al., 2013). We did not, however, observe any upregulation of *Kiss1* or *Tac2* in our *Eed-cKO* scRNA-seq data (Table S2), but rather the loss of a distinct cluster of *Tac2*-expressing cells. These findings indicate that the epigenomic profile of the *Kiss1* and *Tac2* promoters and their sensitivity to PRC2 may change from embryogenesis to early adult life, and from ‘baseline’ to elevated levels of expression.

Finally, in humans, extensive genome and exome sequencing indicates that all major PRC2 components, including *EED*, *EZH2* and *SUZ12* are haploinsufficient (Karczewski et al., 2020). In line with this, a group of related human developmental overgrowth syndromes, including the Weaver, Weaver-like, Cohen-Gibson, and Overgrowth and Intellectual Disability syndromes, appear to be caused by heterozygous mutations in *EED*, *EZH2* or *SUZ12* (reviewed by Burkardt et al., 2019; Cyrus et al., 2019). These syndromes manifest with peripheral overgrowth, as well as neurological defects and intellectual disability. Our finding of a particular sensitivity of DA, Hcrt and Tac2-Pax6 neurons to loss of PRC2 activity may suggest that behaviours related to these hypothalamic cell types may be important to assess in the human PRC2 syndromes. Moreover, the strong links between PRC2 and CNS proliferation, the haploinsufficiency of PRC2 complex genes, and the involvement of PRC2 in overgrowth and intellectual disability syndromes, may have bearing upon adult neurogenesis in the hypothalamus (reviewed by Cheng, 2013; Yoo and Blackshaw, 2018).

## MATERIALS AND METHODS

### Mouse stocks

The *Eed^fl/fl^* allele has exons 3-6 flanked by *loxP* sites (Xie et al., 2014), and was obtained from the Jackson Laboratory Stock Center (stock number 022727). The *Sox1-Cre* line (Takashima et al., 2007) was provided by Jose Dias and Johan Ericson (Karolinska Institute, Stockholm, Sweden). Stocks were maintained on a *B6:129S1* mixed background. Mice were maintained at Linkoping University animal facility, in accordance with best practices. All mouse procedures were approved by the regional animal ethics committee Linkopings djurforsoksetiska namnd (Dnr 69-14). The morning of the vaginal plug was set as 0.5 days post coitum, i.e. E0.5. Pregnant females were sacrificed, and embryos dissected at E12.5, E13.5, E15.5, E16.5 and E18.5. Primers for genotyping were: *Cre1*: GCGGTCTGGCAGTAAAAACTATC; *Cre2*: GTGAAACAGCATTGCTGTCACTT; *Eed1*: GGGACGTGCTGACATTTTCT; *Eed2*: CTTGGGTGGTTTGGCTAAGA; male/female primers (Clapcote and Roder, 2005) forward: CTGAAGCTTTTGGCTTTGAG; reverse: CCACTGCCAAATTCTTTGG.

### Immunohistochemistry

Mouse embryos were dissected to extract the brains, which were fixed in 4% paraformaldehyde (PFA) at 4°C for 18-36 h and kept in 30% sucrose at 4°C until saturated, upon which they were frozen in OCT Tissue Tek (Sakura Finetek) and stored at −80°C. Cryosections (30 µm) were treated with 4% PFA for 15 min at room temperature, blocked and processed with primary antibodies in PBS with 0.2% Triton X-100 and 4% horse serum overnight at 4°C. Secondary antibodies, conjugated with AMCA, FITC, Rhodamine-RedX, Cy5 (Jackson ImmunoResearch), or AFD555, AF568 or AF647 (Thermo Fisher Scientific) were used at 1:200. Slides were mounted in Vectashield (Vector Laboratories). DAPI was included in the secondary antibody solution. Primary antibodies used were: goat anti-Sox2 (1:250, SC-17320, Santa Cruz Biotechnology); rabbit anti-H3K27me3 (1:500, 9733, Cell Signaling Technology); rat anti-PH3-Ser28 (1:1000; ab10543, Abcam); rabbit anti-Pax2 (1:100, ab232460, Abcam), rabbit anti-TFAP2b (1:100, #ab221094, Abcam), rabbit anti-Orexin A (Hcrt) (1:1000, ab6214, Abcam), rabbit anti-Avp (1:500, #ab213708, Abcam).

### Confocal imaging and data acquisition

Fluorescent images were obtained with Zeiss LSM700 or Zeiss LSM800 confocal microscopes. Confocal stacks were merged using Fiji software (Schindelin et al., 2012). Compilation of images and graphs was carried out using Adobe Illustrator.

### Quantification of proliferation

On confocal images of sagittal sections, a 400 µm wide (segmented line) selection was made along the rim of the hypothalamic tissue using ImageJ (Fiji) software. This selection was straightened. A second subselection of 1000 µm in length of the straightened tissue was made. The final tissue selection analyzed was 400×1000 µm. PH3, DAPI and Sox2 staining were quantified using Fiji software (Schindelin et al., 2012, 2015). Then 3D reconstruction and volume quantification of DAPI and Sox2 signals were achieved using the 3DViewer Fiji plugin (Schmid et al., 2010) in the selected regions, considering the anatomical features and excluding non-CNS tissue. Mitotic cells (PH3+) were counted in the selected region. Proliferation analyses are presented as ratios of mitotic cells to DAPI or Sox2 calculated volumes within the selected regions. ImageJ (Fiji) thresholding methods (functions) ‘Huang’ & ‘Moments’ were used to remove background/noise from signal in volumetrics analysis of Sox2 and DAPI, respectively. For mitotic analyses, unpaired two-tailed Student's *t*-test was performed (*P≤0.05, **P≤0.01 or ***P≤0.001). Microsoft Excel 2010 and GraphPad Prism 8.3.0 were used for statistical analyses, data compilation and graphical representation. See Table S8 for further details.

### scRNA-seq

#### Data generation

E12.5, E13.5, E15.5, E16.5 and E18.5 embryos (control: E12.5, E13.5, E15.5, E16.5 and E18.5; *Eed-cKO*: E13.5, E15.5 and E18.5) embryos were harvested and genotyped. The hypothalamus was dissected out in ice-cold RPMI-1640 medium (11530586, Thermo Fisher Scientific). The dissected hypothalami were further divided into 2-4 pieces before cell dissociation for single cell isolation using Papain Dissociation System (LK003150, Worthington Biochemical Corporation). The tissues were incubated with papain solution at 37°C for 60 min, under slow shaking (80 rpm). Isolated single cells were re-suspended in ice-cold 1×PBS, 0.1% bovine serum albumin, to a final concentration of 2500 cells/μl (±10%). Cells were checked for viability using a Bio-Rad TC10 automated cell counter. Single cells and barcode beads were encapsulated into droplets using Bio-Rad ddSEQ Single-Cell Isolator for cell lysis and barcoding (12004336). Subsequently, RNA-seq libraries were generated using SureCell WTA 3′ Library Prep Kit for the ddSEQ System (six cartridge version, 20014280, Illumina). Libraries were assessed for quality, using 1 µl of undiluted cDNA, on an Agilent Technology 2100 Bioanalyzer, using an Agilent High Sensitivity DNA chip (5067-4626) to determine fragment size and yield. Samples were stored at −20°C until sequencing. Libraries were normalized and sequenced in pools of four, whereby libraries prepared on the same ddSEQ cartridge were balanced between different library pools to control for batch effects. The libraries were sequenced on an Illumina NextSeq500 system, using NextSeq 500/550 High Output Kit v2.5 (150 cycles; 20024907), paired-end read type and Single-Index. This platform does not have physically separate sequencing lanes, and thus pooled samples were sequenced over all four lanes of the NextSeq flow cell. The scRNA-seq fastq files were generated using the Illumina Base Space application *FASTQ Generation* v1.0.0. The scRNA-seq fastq files are available at GEO (GSE154995 for E18.5 and GSE167921 for other stages). Labels and UMAP coordinates of individual cells shown throughout are provided in Table S12.

#### Cell count matrix generation and quality control

Cell UMI counts were generated using the Illumina Base Space application SureCell RNA Single-Cell v1.2.0, which uses STAR (v2.5.2b) to align the cDNA reads to the mm10 genome (the second read mate, R2) and samtools (v1.3) to append cell barcodes to aligned reads (derived from the first read mate, R1). The generated counts for each sequencing run in each sample were concatenated into a single count matrix. For technical quality control, cells with less than 600 UMIs, less than 1.2 UMIs/expressed gene and less than 300 genes expressed were removed (Figs S3 and S12).

We then performed biological filtering to remove cells which do not develop within the hypothalamus. For these steps, we used ∼500,000 reference single cell expression profiles with cell type labels (TaxonomyRank4 labels) from the mouse nervous system (http://mousebrain.org/downloads.html) (Zeisel et al., 2015), in combination with scmapCluster from scmap v1.4.1, to obtain initial cell type labels. Cells that do not develop within the hypothalamus were filtered, which included vasculature and immuno-related cell types (vascular endothelial, vascular/leptomeningial, pericytes, microglia). Normalisation was performed using scanpy v1.8.1 by converting counts to log-counts-per-million (CPM) values after filtering for genes expressed in fewer than three cells.

#### Dimensionality reduction

The main goal of our dimensionality reduction procedure was to capture a joint space of the control and *Eed-cKO* cells that would allow direct comparison of cellular diversity. Toward this end, we used principal components analysis (PCA) for initial dimensionality reduction. PCA requires the input data to be mean-centred and variance-normalised per gene. To satisfy these requirements, we mean-centred and scaled to unit variance our normalised gene expression matrix using the scale function in scikit-learn v0.21.3 before any PCA transformation. Importantly, we then derived the PCA transformation on the control cells alone, and then applied the same transformation on the *Eed-cKO* cells. This ensured that our dimensionality reduction captured the main dimensions of variation in the control cells and not sources of variation due to the *Eed-cKO* treatment, therefore allowing a direct comparison between these two conditions without complicated batch correction techniques. The PCA spaces of the two sets of cells were then concatenated, and UMAP was performed on this joint space using umap-learn v0.3.8. The genes used for each PCA before UMAP embedding are provided in Table S1; these were selected depending on the set of cells analyzed, as detailed below.

#### Gene selection

To prioritise the biological variation, we were interested in – primarily neurotransmitter, neuropeptidergic and DA-ergic diversity – we curated lists of marker genes for such populations. To counteract technical noise from using few genes we selected additional genes based on their correlation with these marker genes (as detailed for the specific analyses below). This approach was inspired by the method Garnett, which performs similar analyses for cell type prediction (Pliner et al., 2019). As evidenced by our resulting clustering and UMAP embeddings, this approach successfully allowed us to analyse hypothalamic cellular diversity in our control and *Eed-cKO* mice.

#### E18.5 all-hypo cells analysis

To investigate the effect of *Eed-cKO* on broad hypothalamic cell types, we compiled a list of marker genes for neurons (GABAergic, glutamatergic) and glia (oligodendrocytes, astrocytes, tanycytes, ependymal cells). These are provided in Table S1. We subsequently labelled cells according to their type based on which marker gene they expressed the most highly. Cells not found to express any of these marker genes but expressing NP-DA markers were labelled as ‘other-neuron’. Cells not expressing any differentiated cell type marker were considered to be un-labelled.

We then selected additional genes which would maximally separate our cell type labels using normalised mutual information (NMI; scikit-learn v0.21.3), a measure of correlation between discretely labelled data. To obtain discrete labels for gene expression, we classified genes as ‘on’ (counts>0), or ‘off’ (counts=0). We then selected the top 300 genes with the highest NMI (see Table S1) between the binary expression of that gene and the cell type labels. Using these marker genes, the dimensionality reduction procedure was performed as detailed above using scanpy v1.8.1 default parameters for UMAP.

#### E18.5 NP-DA cells analysis

Cells were considered NP-DA if they expressed any neuropeptidergic or DA-ergic marker genes compiled from the literature (Table S1). Hypothalamic NP-DA cells were isolated from the rest of the cells before application of dimensionality reduction (detailed above) based on the NP-DA marker genes, along with an additional 93 genes that had the highest Pearson correlation coefficient with at least one of the marker genes. The UMAP was performed based on the top 60 principal components using 20 nearest neighbours, a minimum distance of 0.01, a spread of 3, and the Manhattan distance metric.

#### E18.5 NP-DA cell clustering and cluster differential gene expression

Within the UMAP space containing both the control and *Eed-cKO* NP-DA cells, a nearest neighbour graph was constructed using the kneighbours_graph function in sklearn, with four neighbours and the Euclidean distance measure. The cells were subsequently clustered using the Leiden algorithm (leidenalg v0.8.2) with a resolution of 2. This resulted in 149 clusters. To derive the meaning behind the clustering, we used DE to label clusters according to the combination of NP-DA markers they upregulated. Significantly DEGs were determined for each cluster using scanpy, with a one-versus-rest mode of comparison.

In cases where no NP-DA marker was found to particularly distinguish that cluster, clusters were labelled with the NP-DA markers with the highest proportion of expression in that cluster along with the best distinguishing gene in the extended list of genes used to derive the NP-DA UMAP (Table S1). Clusters were subsequently merged if they were found to upregulate the same combination of NP-DA marker genes and were neighbours in the UMAP space, or the cluster was not found to upregulate any marker but was spatially close in the UMAP space to another cluster and showed clear visual expression of the same marker gene.

This manual curation resulted in 79 hypothalamic NP-DA clusters. The aforementioned method for calling DEGs was subsequently re-run to call DEGs within clusters to compare *Eed-cKO* and control cells (Table S6). The same approach was also taken for the DE comparing the glut-GABA cells within control or *Eed-cKO* against glutamatergic or GABAergic cells (Table S4).

The heatmaps were constructed by taking the average expression of each gene in each NP-DA cluster segmented by control and *Eed-cKO*. The average expression of each gene across the NP-DA clusters was scaled between 0 and 1 to visualise the expression in each cluster relative to the total across the clusters. Scaling was performed to enable direct comparison between the control and *Eed-cKO* heatmaps. These visualisations were constructed using seaborn v0.11.0.

To compare between fast neurotransmitter status [glutamatergic (*Slc17a6*), GABAergic (*Slc32a1*), Glut/GABA (*Slc17a6*-*Slc32a1*)] and NP-DA cell types, we used clustree (Zappia and Oshlack, 2018) v0.4.3 to visualise the relationship between these two sets of cell labels; with connections between the labels drawn if at least 25% of the cells in one grouping overlapped with the other.

#### E18.5 broad differential expression and gene set enrichment

Differential expression of *Eed-cKO* versus control cells – regardless of cluster – was performed using limma-voom (Law et al., 2014) and a pseudobulking approach previously shown to reduce the false positive rate of differential expression in scRNA-seq data (Lun and Marioni, 2017). Pseudobulking involves summing the gene counts across cells captured in an independent sample and then performing differential expression using methods designed for bulk RNA-seq to call DEGs between samples from different conditions. For the E18.5 *Eed-cKO* versus control comparison, we performed two different levels of differential expression; one where samples were pseudobulked using only All-Hypo cells, the other using only NP-DA cells. This ensured the pseudobulking strategy did not average out more specific effects by considering different subpopulations of cells (Table S2). After pseudobulking the counts per sample, we then normalised the data using log-cpm in EdgeR (Robinson et al., 2010) with size factors estimated using the trimmed-mean squares method (Robinson and Oshlack, 2010) after filtering out genes with less than 15 counts detected across samples for All-Hypo, and less than nine counts across samples for NP-DA. Genes with a maximum count smaller than two, and with a median log-cpm expression smaller than 1.5 for the All-Hypo comparison and smaller than 0.45 for the NP-DA comparison, were filtered from subsequent analysis. Relative log expression values were used to confirm appropriate normalisation and assumptions were met for downstream limma-voom differential expression (Gandolfo and Speed, 2018). DEGs with a log-fold-change greater than 0.18 were then called using the TREAT criterion by contrasting *Eed-cKO* samples against control samples after voom mean-variance correction (McCarthy and Smyth, 2009).

Violin plot visualisations of the DEGs only included 5% of zeros for each grouping of cells, to prevent most of the density falling at zero. In the construction of the ‘Transcription Factor’ violin plots, we display the top upregulated genes in the *Eed-cKO* that appear in the GO term ‘DNA-binding transcription factor activity’ (GO:0003700). The violin plots were constructed using seaborn v0.11.0.

To investigate the biological significance of the DEGs between *Eed-cKO* and control, we ranked significantly DEGs by *t*-value from the limma-voom analysis and performed GSEA using clusterProfiler v4.0.0 (Wu et al., 2021) against the Molecular Signature Database (MSigDB) against the Hallmark gene sets and the GO biological process database. The method used was fast GSEA with 10,000 permutations. Terms with adjusted *P*-values smaller than 0.05 were considered significant.

To investigate whether genes which were differentially expressed between *Eed-cKO* and control had commonalities in terms of epigenetics and molecular function, we performed gene set enrichment using the Enrichr method, grouping the genes into up- or downregulated genes using gseapy v0.9.16 (Fig. S4). We tested for enrichment using the GO_Molecular_Function_2018 and the ENCODE_Histone_Modifications_2015 gene sets, thus giving insight into the molecular function and the epigenetic modifications of these genes across tissues (adjusted *P*≤0.5; Table S3).

When visualising these results, we grouped terms based on similarity and counted the number of genes associated with each grouping (Fig. S4C). Terms were grouped as ‘TF activity/DNA binding’ if they mentioned ‘binding’ or ‘transcription’. Terms were grouped as ‘H3K27me3’, or ‘H3K4me1/3’ if these epigenetic marks were mentioned in the term name. The count of DEGs which appeared in terms belonging to each combination of groupings is shown (Fig. S4C). Term groupings were based on a manual inspection of the enriched terms which revealed an over-representation of terms related to the aforementioned groupings (Table S3).

#### Differential abundance analysis and doublet detection

To call differential abundance in the ratio of cell types detected between samples of *Eed-cKO* and control, we took a compositional data analysis (CoDA) approach, whereby transformations on ratios are performed to convert from the geometry of the simplex to standard Euclidean space (Quinn et al., 2019). This conversion is necessary as ratios are inherently constrained to values between 0 and 1 (simplex geometry), and so standard statistical techniques cannot be appropriately applied as these do not account for the restricted value range (Quinn et al., 2019). Ratios of observed cells of a particular type in a sample were subsequently transformed using the centred-log-ratio (CLR; scikit-bio v0.5.6) followed by independent *t*-tests (scipy v1.6.2) comparing the CLRs between *Eed-cKO* and control samples of the indicated cell type in the results. Pseudocounts of 0.1 were added to the counts of samples where no cells of a particular labelling were detected.

For doublet detection, we ran DoubletFinder v2.0.3 (McGinnis et al., 2019) using all cells that passed quality control on each sample. DoubletFinder uses random sampling of cells to create artificial doublets and then calculates the proportion of artificial nearest neighbours for each real cell to predict doublets. Before application of DoubletFinder, on each sample we performed basic pre-processing using Seurat v4.0.5 (Hao et al., 2021), which included: log-cpm normalisation, finding the top 2000 variable genes with the variance stabilising transformation method, scaling the data and then running PCA followed by UMAP on the top 10 principal components. We then selected the optimal pK value (principal component neighbourhood size) by taking the maximum value at which the mean-variance normalised binomial coefficient (BCmvn) is maximised. Doublets were then predicted with a 6% doublet rate per sample using the top 10 principal components to measure the proportion of artificial nearest neighbours. Differential abundance analysis as described above was performed with and without the doublets removed on the glut-GABA cells to show that the increased numbers of these cells were not due to doublets (Fig. S13).

#### Early time point cell labelling and dimensionality reduction

After quality control, as previously described, we subsequently labelled cells based on expression of known marker genes for glutamatergic, GABAergic, glial, and ventricular zone and subventricular zone cell types (the full cell type and marker list can be seen in Table S13). Due to varying specificity of markers for their respective cell types, we marked cells as belonging to a particular group in a hierarchical manner to alleviate this problem; whereby least-specific markers were used to mark cells first, and most-specific markers to mark cells last such that prior labelling was overridden. The order this was performed is also shown in Table S13, where groups labelled first to last are listed from the top row of the table to the bottom row of the table.

A gene was considered ‘on’ in a particular cell if that cell expressed the gene in the upper 40th percentile of the non-zero gene expression range of that gene. Labelling of cells was performed based on this binarized (‘on’/‘off’) transformation of the gene expression in each cell. In the labelling process, we also considered negative gene expression indicators for particular cell types, and also ‘or’ and ‘and’ logic, where cells could be considered of a certain type if they expressed/did not express indicated marker genes in part or in combination. The exact logical notation used in each case is provided in Table S13.

As a further contingency to prevent labelling cells incorrectly and to reduce the number of unlabelled cells, we then trained a logistic regression classifier (as implemented in sklearn v0.22.2) using all the genes present in Table S13 as well as all mouse transcription factors (as listed in GO term GO:0003700). To train the classifier on relevant genes with this set, we selected the top 300 genes that, when considering their binarized expression (expression>0), had the highest NMI score with a randomly selected 80% of cells labelled using marker genes as a training set. We then trained the logistic regression classifier on this training set using the L1 penalty and liblinear solver, and then predicted the labels for the remaining 20% of binary labelled cells and the unlabelled cells. If the probability of the most likely predicted label for a given cell was smaller than 0.5, the cell was labelled ‘unknown’. The confusion matrix for the left out 20% of cells is presented in Table S13, showing the labelling approach taken was effective in labelling the numerous kinds of broad cell types.

We performed dimensionality reduction as described above, except the gene set used was the top 300 most highly informative genes used for training the logistic regression classifier for the early time point cell labelling. The UMAP was then performed by creating a nearest-neighbours graph using scanpy v1.6.1 with ten neighbours and the top 40 principal components, followed by scanpy's UMAP function applied with default settings.

#### Ependymal and tanycyte trajectory analysis

To recreate the previously observed lineage trajectory of ependymal and tanycytes in both our control and *Eed-cKO* cells (Kim et al., 2020), we first isolated all cells from the E13.5, E15.5, and E18.5 data after technical and biological filtering that expressed either *Foxj1*, *Rax* or *Wnt7*, gene markers for ependymal cells, tanycytes and their progenitors, respectively. We then filtered genes with a minimum number of five counts across all cells and a minimum number of three cells. Control and *Eed-cKO* cells were then integrated to the same joint space using scanorama (Hie et al., 2019) with default parameters, followed by the following calculations on the scanorama joint space with scanpy (Wolf et al., 2018): nearest neighbour determination, Leiden clustering and UMAP dimensionality reduction. Clusters with clearly high expression of *Foxj1* and *Rax* which were connected by a highly expressing *Wnt7b* cluster were then isolated for trajectory inference with PAGA (Wolf et al., 2019). Comparing the trends of previously identified markers of different stages of the tanycyte/ependymal bifurcation revealed a striking similarity when separating cells into control and *Eed-cKO* within the inferred lineage trajectory (Fig. S14).

#### Early time point differential expression

With the generation of samples from additional time points E13.5 and E15.5 for both *Eed-cKO* and control, we performed DE analysis with the same approach using all cells that passed quality control, with the following exceptions: we filtered genes from analysis that had a maximum number of counts across samples less than ten and a median log-cpm smaller than 1, and the design matrix reflected the more complex experimental design (Eqn 1).
(1)

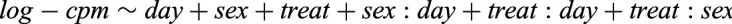
The greater detail in the experimental design allowed us to explore broad differential expression between *Eed-cKO* and control while controlling for other factors (Fig. S4D; Table S2) including: the interaction effect between time point and *Eed-cKO* (Fig. S4G; Table S2), differential expression between sex (Fig. S4H), and the interaction effect between *Eed-cKO* and sex (Fig. S4I).

#### Early time point identification of *Eed-cKO* affected genes in Lhx9 and Glut/GABA cells

We first called DEGs between control and *Eed-cKO* within the groups of Lhx9 and Glut/GABA cells using a pseudosampling approach followed by a *t*-test to call DEGs. Namely, we created 20 random samples of approximately equal numbers of cells without replacement within each group/condition combination. The gene expression of these pseudosampled groups were then averaged, and DEGs were called between *Eed-cKO* and control using a *t*-test (scanpy) with each pseudosample as an observation. Genes with a false discovery rate adjusted *P*-value <0.05 were considered significant (Table S11).

The pseudosampling approach was then repeated to compare *Eed-cKO* cells from Lhx9 and Glut/GABA cells with all other *Eed-cKO* cells (Table S11). To identify the *Eed-cKO* genes that were specifically upregulated in the *Eed-cKO* Lhx9 and/or Glut/GABA cells, we then took the overlap between the upregulated genes from comparing Lhx9 and Glut/GABA *Eed-cKO* versus Lhx9 and Glut/GABA control cells, and the DEGs from comparing Lhx9/Glut/GABA *Eed-cKO* cells versus all other *Eed-cKO* cells (Table S11).

## Supplementary Material

Supplementary information

## References

[DEV200076C1] Alpár, A., Benevento, M., Romanov, R. A., Hökfelt, T. and Harkany, T. (2019). Hypothalamic cell diversity: non-neuronal codes for long-distance volume transmission by neuropeptides. *Curr. Opin. Neurobiol.* 56, 16-23. 10.1016/j.conb.2018.10.012 30471413

[DEV200076C2] Alvarez-Bolado, G. (2019). Development of neuroendocrine neurons in the mammalian hypothalamus. *Cell Tissue Res.* 375, 23-39. 10.1007/s00441-018-2859-1 29869716

[DEV200076C3] Arcipowski, K. M., Martinez, C. A. and Ntziachristos, P. (2016). Histone demethylases in physiology and cancer: a tale of two enzymes, JMJD3 and UTX. *Curr. Opin. Genet. Dev.* 36, 59-67. 10.1016/j.gde.2016.03.010 27151432 PMC4880520

[DEV200076C4] Ashburner, M., Ball, C. A., Blake, J. A., Botstein, D., Butler, H., Cherry, J. M., Davis, A. P., Dolinski, K., Dwight, S. S., Eppig, J. T. et al. (2000). Gene ontology: tool for the unification of biology. The Gene Ontology Consortium. *Nat. Genet.* 25, 25-29. 10.1038/75556 10802651 PMC3037419

[DEV200076C5] Aylwin, C. F., Vigh-Conrad, K. and Lomniczi, A. (2019). The emerging role of chromatin remodeling factors in female pubertal development. *Neuroendocrinology* 109, 208-217. 10.1159/000497745 30731454 PMC6794153

[DEV200076C6] Bahrampour, S., Jonsson, C. and Thor, S. (2019). Brain expansion promoted by polycomb-mediated anterior enhancement of a neural stem cell proliferation program. *PLoS Biol.* 17, e3000163. 10.1371/journal.pbio.3000163 30807568 PMC6407790

[DEV200076C7] Banaszynski, L. A., Wen, D., Dewell, S., Whitcomb, S. J., Lin, M., Diaz, N., Elsässer, S. J., Chapgier, A., Goldberg, A. D., Canaani, E. et al. (2013). Hira-dependent histone H3.3 deposition facilitates PRC2 recruitment at developmental loci in ES cells. *Cell* 155, 107-120. 10.1016/j.cell.2013.08.061 24074864 PMC3838450

[DEV200076C8] Bedont, J. L., Newman, E. A. and Blackshaw, S. (2015). Patterning, specification, and differentiation in the developing hypothalamus. *Wiley Interdiscip. Rev. Dev. Biol.* 4, 445-468. 10.1002/wdev.187 25820448 PMC5890958

[DEV200076C9] Biran, J., Tahor, M., Wircer, E. and Levkowitz, G. (2015). Role of developmental factors in hypothalamic function. *Front. Neuroanat.* 9, 47. 10.3389/fnana.2015.00047 25954163 PMC4404869

[DEV200076C10] Björklund, A. and Dunnett, S. B. (2007). Dopamine neuron systems in the brain: an update. *Trends Neurosci.* 30, 194-202. 10.1016/j.tins.2007.03.006 17408759

[DEV200076C11] Blackshaw, S., Scholpp, S., Placzek, M., Ingraham, H., Simerly, R. and Shimogori, T. (2010). Molecular pathways controlling development of thalamus and hypothalamus: from neural specification to circuit formation. *J. Neurosci.* 30, 14925-14930. 10.1523/JNEUROSCI.4499-10.2010 21068293 PMC6633850

[DEV200076C12] Blanco, E., González-Ramírez, M., Alcaine-Colet, A., Aranda, S. and Di Croce, L. (2020). The bivalent genome: characterization, structure, and regulation. *Trends Genet.* 36, 118-131. 10.1016/j.tig.2019.11.004 31818514

[DEV200076C13] Burbridge, S., Stewart, I. and Placzek, M. (2016). Development of the neuroendocrine hypothalamus. *Compr. Physiol.* 6, 623-643. 10.1002/cphy.c150023 27065164

[DEV200076C14] Burgold, T., Spreafico, F., De Santa, F., Totaro, M. G., Prosperini, E., Natoli, G. and Testa, G. (2008). The histone H3 lysine 27-specific demethylase Jmjd3 is required for neural commitment. *PLoS ONE* 3, e3034. 10.1371/journal.pone.0003034 18716661 PMC2515638

[DEV200076C15] Burkardt, D. D., Tatton-Brown, K., Dobyns, W. and Graham, J. M. Jr. (2019). Approach to overgrowth syndromes in the genome era. *Am. J. Med. Genet. C Semin. Med. Genet.* 181, 483-490. 10.1002/ajmg.c.31757 31793186

[DEV200076C16] Campbell, J. N., Macosko, E. Z., Fenselau, H., Pers, T. H., Lyubetskaya, A., Tenen, D., Goldman, M., Verstegen, A. M. J., Resch, J. M., McCarroll, S. A. et al. (2017). A molecular census of arcuate hypothalamus and median eminence cell types. *Nat. Neurosci.* 20, 484-496. 10.1038/nn.4495 28166221 PMC5323293

[DEV200076C17] Caviness, V. S., Jr, Takahashi, T. and Nowakowski, R. S. (1995). Numbers, time and neocortical neuronogenesis: a general developmental and evolutionary model. *Trends Neurosci.* 18, 379-383. 10.1016/0166-2236(95)93933-O 7482802

[DEV200076C18] Chammas, P., Mocavini, I. and Di Croce, L. (2020). Engaging chromatin: PRC2 structure meets function. *Br. J. Cancer* 122, 315-328. 10.1038/s41416-019-0615-2 31708574 PMC7000746

[DEV200076C19] Chen, R., Wu, X., Jiang, L. and Zhang, Y. (2017). Single-cell RNA-seq reveals hypothalamic cell diversity. *Cell Rep.* 18, 3227-3241. 10.1016/j.celrep.2017.03.004 28355573 PMC5782816

[DEV200076C20] Cheng, M.-F. (2013). Hypothalamic neurogenesis in the adult brain. *Front. Neuroendocrinol.* 34, 167-178. 10.1016/j.yfrne.2013.05.001 23684668

[DEV200076C21] Cheng, L., Arata, A., Mizuguchi, R., Qian, Y., Karunaratne, A., Gray, P. A., Arata, S., Shirasawa, S., Bouchard, M., Luo, P. et al. (2004). Tlx3 and Tlx1 are post-mitotic selector genes determining glutamatergic over GABAergic cell fates. *Nat. Neurosci.* 7, 510-517. 10.1038/nn1221 15064766

[DEV200076C22] Clapcote, S. J. and Roder, J. C. (2005). Simplex PCR assay for sex determination in mice. *BioTechniques* 38, 702, 704, 706. 10.2144/05385BM05 15945368

[DEV200076C23] Coleman, R. T. and Struhl, G. (2017). Causal role for inheritance of H3K27me3 in maintaining the OFF state of a Drosophila HOX gene. *Science* 356, eaai8236. 10.1126/science.aai8236 28302795 PMC5595140

[DEV200076C24] Cyrus, S., Burkardt, D., Weaver, D. D. and Gibson, W. T. (2019). PRC2-complex related dysfunction in overgrowth syndromes: a review of EZH2, EED, and SUZ12 and their syndromic phenotypes. *Am. J. Med. Genet. C Semin. Med. Genet.* 181, 519-531. 10.1002/ajmg.c.31754 31724824

[DEV200076C25] Dalal, J., Roh, J. H., Maloney, S. E., Akuffo, A., Shah, S., Yuan, H., Wamsley, B., Jones, W. B., de Guzman Strong, C., Gray, P. A. et al. (2013). Translational profiling of hypocretin neurons identifies candidate molecules for sleep regulation. *Genes Dev.* 27, 565-578. 10.1101/gad.207654.112 23431030 PMC3605469

[DEV200076C26] Davis, C. A., Hitz, B. C., Sloan, C. A., Chan, E. T., Davidson, J. M., Gabdank, I., Hilton, J. A., Jain, K., Baymuradov, U. K., Narayanan, A. K. et al. (2018). The Encyclopedia of DNA elements (ENCODE): data portal update. *Nucleic Acids Res.* 46, D794-D801. 10.1093/nar/gkx1081 29126249 PMC5753278

[DEV200076C27] De La Herran-Arita, A. K., Zomosa-Signoret, V. C., Millan-Aldaco, D. A., Palomero-Rivero, M., Guerra-Crespo, M., Drucker-Colin, R. and Vidaltamayo, R. (2011). Aspects of the narcolepsy-cataplexy syndrome in O/E3-null mutant mice. *Neuroscience* 183, 134-143. 10.1016/j.neuroscience.2011.03.029 21435382

[DEV200076C28] Economides, K. D., Zeltser, L. and Capecchi, M. R. (2003). Hoxb13 mutations cause overgrowth of caudal spinal cord and tail vertebrae. *Dev. Biol.* 256, 317-330. 10.1016/S0012-1606(02)00137-9 12679105

[DEV200076C29] Faust, C., Schumacher, A., Holdener, B. and Magnuson, T. (1995). The eed mutation disrupts anterior mesoderm production in mice. *Development (Cambridge, England)* 121, 273-285. 10.1242/dev.121.2.273 7768172

[DEV200076C30] Faust, C., Lawson, K. A., Schork, N. J., Thiel, B. and Magnuson, T. (1998). The Polycomb-group gene eed is required for normal morphogenetic movements during gastrulation in the mouse embryo. *Development* 125, 4495-4506. 10.1242/dev.125.22.4495 9778508

[DEV200076C31] Ferran, J. L., Puelles, L. and Rubenstein, J. L. R. (2015). Molecular codes defining rostrocaudal domains in the embryonic mouse hypothalamus. *Front. Neuroanat.* 9, 46. 10.3389/fnana.2015.00046 25941476 PMC4400913

[DEV200076C32] Gandolfo, L. C. and Speed, T. P. (2018). RLE plots: visualizing unwanted variation in high dimensional data. *PLoS ONE* 13, e0191629. 10.1371/journal.pone.0191629 29401521 PMC5798764

[DEV200076C33] Gene Ontology Consortium. (2021). The Gene Ontology resource: enriching a GOld mine. *Nucleic Acids Res.* 49, D325-D334. 10.1093/nar/gkaa1113 33290552 PMC7779012

[DEV200076C34] Hao, Y., Hao, S., Andersen-Nissen, E., Mauck, W. M., III, Zheng, S., Butler, A., Lee, M. J., Wilk, A. J., Darby, C., Zager, M. et al. (2021). Integrated analysis of multimodal single-cell data. *Cell* 184, 3573-3587.e29. 10.1016/j.cell.2021.04.048 34062119 PMC8238499

[DEV200076C35] Henry, F. E., Sugino, K., Tozer, A., Branco, T. and Sternson, S. M. (2015). Cell type-specific transcriptomics of hypothalamic energy-sensing neuron responses to weight-loss. *eLife* 4, e09800. 10.7554/eLife.09800 PMC4595745 26329458

[DEV200076C36] Hie, B., Bryson, B. and Berger, B. (2019). Efficient integration of heterogeneous single-cell transcriptomes using Scanorama. *Nat. Biotechnol.* 37, 685-691. 10.1038/s41587-019-0113-3 31061482 PMC6551256

[DEV200076C37] Huisman, C., Cho, H., Brock, O., Lim, S. J., Youn, S. M., Park, Y., Kim, S., Lee, S.-K., Delogu, A. and Lee, J. W. (2019). Single cell transcriptome analysis of developing arcuate nucleus neurons uncovers their key developmental regulators. *Nat. Commun.* 10, 3696. 10.1038/s41467-019-11667-y 31420539 PMC6697706

[DEV200076C38] Huisman, C., Kim, Y. A., Jeon, S., Shin, B., Choi, J., Lim, S. J., Youn, S. M., Park, Y., K, C. M., Kim, S. et al. (2021). The histone H3-lysine 4-methyltransferase Mll4 regulates the development of growth hormone-releasing hormone-producing neurons in the mouse hypothalamus. *Nat. Commun.* 12, 256. 10.1038/s41467-020-20511-7 33431871 PMC7801453

[DEV200076C39] Isono, K.-I., Fujimura, Y.-I., Shinga, J., Yamaki, M., O-Wang, J., Takihara, Y., Murahashi, Y., Takada, Y., Mizutani-Koseki, Y. and Koseki, H. (2005). Mammalian polyhomeotic homologues Phc2 and Phc1 act in synergy to mediate polycomb repression of Hox genes. *Mol. Cell. Biol.* 25, 6694-6706. 10.1128/MCB.25.15.6694-6706.2005 16024804 PMC1190356

[DEV200076C40] Jadhav, U., Manieri, E., Nalapareddy, K., Madha, S., Chakrabarti, S., Wucherpfennig, K., Barefoot, M. and Shivdasani, R. A. (2020). Replicational dilution of H3K27me3 in mammalian cells and the role of poised promoters. *Mol. Cell* 78, 141-151.e5. 10.1016/j.molcel.2020.01.017 32027840 PMC7376365

[DEV200076C41] Jeong, J. H., Woo, Y. J., Chua, S., Jr and Jo, Y.-H. (2016). Single-cell gene expression analysis of cholinergic neurons in the arcuate nucleus of the hypothalamus. *PLoS ONE* 11, e0162839. 10.1371/journal.pone.0162839 27611685 PMC5017726

[DEV200076C42] Karczewski, K. J., Francioli, L. C., Tiao, G., Cummings, B. B., Alföldi, J., Wang, Q., Collins, R. L., Laricchia, K. M., Ganna, A., Birnbaum, D. P. et al. (2020). The mutational constraint spectrum quantified from variation in 141,456 humans. *Nature* 581, 434-443. 10.1038/s41586-020-2308-7 32461654 PMC7334197

[DEV200076C43] Kim, D.-W., Yao, Z., Graybuck, L. T., Kim, T. K., Nguyen, T. N., Smith, K. A., Fong, O., Yi, L., Koulena, N., Pierson, N. et al. (2019). Multimodal analysis of cell types in a hypothalamic node controlling social behavior. *Cell* 179, 713-728.e17. 10.1016/j.cell.2019.09.020 31626771 PMC7534821

[DEV200076C44] Kim, D. W., Washington, P. W., Wang, Z. Q., Lin, S. H., Sun, C., Ismail, B. T., Wang, H., Jiang, L. and Blackshaw, S. (2020). The cellular and molecular landscape of hypothalamic patterning and differentiation from embryonic to late postnatal development. *Nat. Commun.* 11, 4360. 10.1038/s41467-020-18231-z 32868762 PMC7459115

[DEV200076C45] Kurrasch, D. M., Cheung, C. C., Lee, F. Y., Tran, P. V., Hata, K. and Ingraham, H. A. (2007). The neonatal ventromedial hypothalamus transcriptome reveals novel markers with spatially distinct patterning. *J. Neurosci.* 27, 13624-13634. 10.1523/JNEUROSCI.2858-07.2007 18077674 PMC6673626

[DEV200076C46] Lam, B. Y. H., Cimino, I., Polex-Wolf, J., Nicole Kohnke, S., Rimmington, D., Iyemere, V., Heeley, N., Cossetti, C., Schulte, R., Saraiva, L. R. et al. (2017). Heterogeneity of hypothalamic pro-opiomelanocortin-expressing neurons revealed by single-cell RNA sequencing. *Mol. Metab.* 6, 383-392. 10.1016/j.molmet.2017.02.007 28462073 PMC5404100

[DEV200076C47] Laprell, F., Finkl, K. and Muller, J. (2017). Propagation of Polycomb-repressed chromatin requires sequence-specific recruitment to DNA. *Science* 356, 85-88. 10.1126/science.aai8266 28302792

[DEV200076C48] Laugesen, A., Højfeldt, J. W. and Helin, K. (2019). Molecular mechanisms directing PRC2 recruitment and H3K27 methylation. *Mol. Cell* 74, 8-18. 10.1016/j.molcel.2019.03.011 30951652 PMC6452890

[DEV200076C49] Law, C. W., Chen, Y., Shi, W. and Smyth, G. K. (2014). voom: Precision weights unlock linear model analysis tools for RNA-seq read counts. *Genome Biol.* 15, R29. 10.1186/gb-2014-15-2-r29 24485249 PMC4053721

[DEV200076C50] Li, X., Isono, K.-I., Yamada, D., Endo, T. A., Endoh, M., Shinga, J., Mizutani-Koseki, Y., Otte, A. P., Casanova, M., Kitamura, H. et al. (2011). Mammalian polycomb-like Pcl2/Mtf2 is a novel regulatory component of PRC2 that can differentially modulate polycomb activity both at the Hox gene cluster and at Cdkn2a genes. *Mol. Cell. Biol.* 31, 351-364. 10.1128/MCB.00259-10 21059868 PMC3019975

[DEV200076C51] Liu, J., Merkle, F. T., Gandhi, A. V., Gagnon, J. A., Woods, I. G., Chiu, C. N., Shimogori, T., Schier, A. F. and Prober, D. A. (2015). Evolutionarily conserved regulation of hypocretin neuron specification by Lhx9. *Development* 142, 1113-1124. 10.1242/dev.117424 25725064 PMC4360184

[DEV200076C52] Lomniczi, A., Loche, A., Castellano, J. M., Ronnekleiv, O. K., Bosch, M., Kaidar, G., Knoll, J. G., Wright, H., Pfeifer, G. P. and Ojeda, S. R. (2013). Epigenetic control of female puberty. *Nat. Neurosci.* 16, 281-289. 10.1038/nn.3319 23354331 PMC3581714

[DEV200076C53] Lun, A. T. L. and Marioni, J. C. (2017). Overcoming confounding plate effects in differential expression analyses of single-cell RNA-seq data. *Biostatistics* 18, 451-464. 10.1093/biostatistics/kxw055 28334062 PMC5862359

[DEV200076C54] McCarthy, D. J. and Smyth, G. K. (2009). Testing significance relative to a fold-change threshold is a TREAT. *Bioinformatics* 25, 765-771. 10.1093/bioinformatics/btp053 19176553 PMC2654802

[DEV200076C55] McGinnis, C. S., Murrow, L. M. and Gartner, Z. J. (2019). DoubletFinder: doublet detection in single-cell RNA sequencing data using artificial nearest neighbors. *Cell Syst.* 8, 329-337.e24. 10.1016/j.cels.2019.03.003 30954475 PMC6853612

[DEV200076C56] Mickelsen, L. E., Kolling, F. W., IV, Chimileski, B. R., Fujita, A., Norris, C., Chen, K., Nelson, C. E. and Jackson, A. C. (2017). Neurochemical heterogeneity among lateral hypothalamic Hypocretin/Orexin and melanin-concentrating hormone neurons identified through single-cell gene expression analysis. *eNeuro* 4, ENEURO.0013-17.2017. 10.1523/ENEURO.0013-17.2017 PMC5617207 28966976

[DEV200076C57] Mickelsen, L. E., Bolisetty, M., Chimileski, B. R., Fujita, A., Beltrami, E. J., Costanzo, J. T., Naparstek, J. R., Robson, P. and Jackson, A. C. (2019). Single-cell transcriptomic analysis of the lateral hypothalamic area reveals molecularly distinct populations of inhibitory and excitatory neurons. *Nat. Neurosci.* 22, 642-656. 10.1038/s41593-019-0349-8 30858605 PMC7043322

[DEV200076C58] Moffitt, J. R., Bambah-Mukku, D., Eichhorn, S. W., Vaughn, E., Shekhar, K., Perez, J. D., Rubinstein, N. D., Hao, J., Regev, A., Dulac, C. et al. (2018). Molecular, spatial, and functional single-cell profiling of the hypothalamic preoptic region. *Science* 362, eaau5324. 10.1126/science.aau5324 30385464 PMC6482113

[DEV200076C59] Monedero Cobeta, I., Salmani, B. Y. and Thor, S. (2017). Anterior-posterior gradient in neural stem and daughter cell proliferation governed by spatial and temporal Hox control. *Curr. Biol.* 27, 1161-1172. 10.1016/j.cub.2017.03.023 28392108

[DEV200076C60] Montgomery, N. D., Yee, D., Chen, A., Kalantry, S., Chamberlain, S. J., Otte, A. P. and Magnuson, T. (2005). The murine polycomb group protein Eed is required for global histone H3 lysine-27 methylation. *Curr. Biol.* 15, 942-947. 10.1016/j.cub.2005.04.051 15916951

[DEV200076C61] Nesan, D. and Kurrasch, D. M. (2016). Genetic programs of the developing tuberal hypothalamus and potential mechanisms of their disruption by environmental factors. *Mol. Cell. Endocrinol.* 438, 3-17. 10.1016/j.mce.2016.09.031 27720896

[DEV200076C62] Orquera, D. P., Nasif, S., Low, M. J., Rubinstein, M. and de Souza, F. S. J. (2016). Essential function of the transcription factor Rax in the early patterning of the mammalian hypothalamus. *Dev. Biol.* 416, 212-224. 10.1016/j.ydbio.2016.05.021 27212025 PMC4961556

[DEV200076C63] Park, D. H., Hong, S. J., Salinas, R. D., Liu, S. J., Sun, S. W., Sgualdino, J., Testa, G., Matzuk, M. M., Iwamori, N. and Lim, D. A. (2014). Activation of neuronal gene expression by the JMJD3 demethylase is required for postnatal and adult brain neurogenesis. *Cell Rep.* 8, 1290-1299. 10.1016/j.celrep.2014.07.060 25176653 PMC4201382

[DEV200076C64] Pevny, L. H., Sockanathan, S., Placzek, M. and Lovell-Badge, R. (1998). A role for SOX1 in neural determination. *Development* 125, 1967-1978. 10.1242/dev.125.10.1967 9550729

[DEV200076C65] Pillai, A., Mansouri, A., Behringer, R., Westphal, H. and Goulding, M. (2007). Lhx1 and Lhx5 maintain the inhibitory-neurotransmitter status of interneurons in the dorsal spinal cord. *Development* 134, 357-366. 10.1242/dev.02717 17166926

[DEV200076C66] Piunti, A. and Shilatifard, A. (2016). Epigenetic balance of gene expression by Polycomb and COMPASS families. *Science* 352, aad9780. 10.1126/science.aad9780 27257261

[DEV200076C67] Pliner, H. A., Shendure, J. and Trapnell, C. (2019). Supervised classification enables rapid annotation of cell atlases. *Nat. Methods* 16, 983-986. 10.1038/s41592-019-0535-3 31501545 PMC6791524

[DEV200076C68] Puelles, L. and Rubenstein, J. L. R. (2015). A new scenario of hypothalamic organization: rationale of new hypotheses introduced in the updated prosomeric model. *Front. Neuroanat.* 9, 27. 10.3389/fnana.2015.00027 25852489 PMC4365718

[DEV200076C69] Quinn, T. P., Erb, I., Gloor, G., Notredame, C., Richardson, M. F. and Crowley, T. M. (2019). A field guide for the compositional analysis of any-omics data. *Gigascience* 8, giz107. 10.1093/gigascience/giz107 31544212 PMC6755255

[DEV200076C70] Robinson, M. D. and Oshlack, A. (2010). A scaling normalization method for differential expression analysis of RNA-seq data. *Genome Biol.* 11, R25. 10.1186/gb-2010-11-3-r25 20196867 PMC2864565

[DEV200076C71] Robinson, M. D., McCarthy, D. J. and Smyth, G. K. (2010). edgeR: a Bioconductor package for differential expression analysis of digital gene expression data. *Bioinformatics* 26, 139-140. 10.1093/bioinformatics/btp616 19910308 PMC2796818

[DEV200076C72] Romanov, R. A., Zeisel, A., Bakker, J., Girach, F., Hellysaz, A., Tomer, R., Alpár, A., Mulder, J., Clotman, F., Keimpema, E. et al. (2017). Molecular interrogation of hypothalamic organization reveals distinct dopamine neuronal subtypes. *Nat. Neurosci.* 20, 176-188. 10.1038/nn.4462 27991900 PMC7615022

[DEV200076C73] Romanov, R. A., Alpár, A., Hökfelt, T. and Harkany, T. (2019). Unified classification of molecular, network, and endocrine features of hypothalamic neurons. *Annu. Rev. Neurosci.* 42, 1-26. 10.1146/annurev-neuro-070918-050414 30735460

[DEV200076C74] Romanov, R. A., Tretiakov, E. O., Kastriti, M. E., Zupancic, M., Häring, M., Korchynska, S., Popadin, K., Benevento, M., Rebernik, P., Lallemend, F. et al. (2020). Molecular design of hypothalamus development. *Nature* 582, 246-252. 10.1038/s41586-020-2266-0 32499648 PMC7292733

[DEV200076C75] Saper, C. B. and Lowell, B. B. (2014). The hypothalamus. *Curr. Biol.* 24, R1111-R1116. 10.1016/j.cub.2014.10.023 25465326

[DEV200076C76] Schindelin, J., Arganda-Carreras, I., Frise, E., Kaynig, V., Longair, M., Pietzsch, T., Preibisch, S., Rueden, C., Saalfeld, S., Schmid, B. et al. (2012). Fiji: an open-source platform for biological-image analysis. *Nat. Methods* 9, 676-682. 10.1038/nmeth.2019 22743772 PMC3855844

[DEV200076C77] Schindelin, J., Rueden, C. T., Hiner, M. C. and Eliceiri, K. W. (2015). The ImageJ ecosystem: an open platform for biomedical image analysis. *Mol. Reprod. Dev.* 82, 518-529. 10.1002/mrd.22489 26153368 PMC5428984

[DEV200076C78] Schmid, B., Schindelin, J., Cardona, A., Longair, M. and Heisenberg, M. (2010). A high-level 3D visualization API for Java and ImageJ. *BMC Bioinformatics* 11, 274. 10.1186/1471-2105-11-274 20492697 PMC2896381

[DEV200076C79] Schumacher, A., Faust, C. and Magnuson, T. (1996). Positional cloning of a global regulator of anterior-posterior patterning in mice. *Nature* 384, 648. 10.1038/384648a0 8984348

[DEV200076C80] Shalev, D. and Melamed, P. (2020). The role of the hypothalamus and pituitary epigenomes in central activation of the reproductive axis at puberty. *Mol. Cell. Endocrinol.* 518, 111031. 10.1016/j.mce.2020.111031 32956708

[DEV200076C81] Shimogori, T., Lee, D. A., Miranda-Angulo, A., Yang, Y., Wang, H., Jiang, L., Yoshida, A. C., Kataoka, A., Mashiko, H., Avetisyan, M. et al. (2010). A genomic atlas of mouse hypothalamic development. *Nat. Neurosci.* 13, 767-775. 10.1038/nn.2545 20436479 PMC4067769

[DEV200076C82] Sokolowski, K., Esumi, S., Hirata, T., Kamal, Y., Tran, T., Lam, A., Oboti, L., Brighthaupt, S.-C., Zaghlula, M., Martinez, J. et al. (2015). Specification of select hypothalamic circuits and innate behaviors by the embryonic patterning gene dbx1. *Neuron* 86, 403-416. 10.1016/j.neuron.2015.03.022 25864637 PMC4484744

[DEV200076C83] Squair, J. W., Gautier, M., Kathe, C., Anderson, M. A., James, N. D., Hutson, T. H., Hudelle, R., Qaiser, T., Matson, K. J. E., Barraud, Q. et al. (2021). Confronting false discoveries in single-cell differential expression. *Nat. Commun.* 12, 5692. 10.1038/s41467-021-25960-2 34584091 PMC8479118

[DEV200076C84] Steffen, P. A. and Ringrose, L. (2014). What are memories made of? How Polycomb and Trithorax proteins mediate epigenetic memory. *Nat. Rev. Mol. Cell Biol.* 15, 340-356. 10.1038/nrm3789 24755934

[DEV200076C85] Struhl, G. (1983). Role of the esc+ gene product in ensuring the selective expression of segment-specific homeotic genes in Drosophila. *J. Embryol. Exp. Morphol.* 76, 297-331. 10.1242/dev.76.1.297 6631324

[DEV200076C86] Struhl, G. and Akam, M. (1985). Altered distributions of Ultrabithorax transcripts in extra sex combs mutant embryos of Drosophila. *EMBO J.* 4, 3259-3264. 10.1002/j.1460-2075.1985.tb04075.x 2419125 PMC554652

[DEV200076C87] Sun, H., Wang, Y., Wang, Y., Ji, F., Wang, A., Yang, M., He, X. and Li, L. (2021). Bivalent regulation and related mechanisms of H3K4/27/9me3 in stem cells. *Stem Cell Rev. Rep.* 18, 165-178. 10.1007/s12015-021-10234-7 34417934

[DEV200076C88] Suzuki, M., Mizutani-Koseki, Y., Fujimura, Y.-I., Miyagishima, H., Kaneko, T., Takada, Y., Akasaka, T., Tanzawa, H., Takihara, Y., Nakano, M. et al. (2002). Involvement of the Polycomb-group gene Ring1B in the specification of the anterior-posterior axis in mice. *Development* 129, 4171-4183. 10.1242/dev.129.18.4171 12183370

[DEV200076C89] Takahashi, T., Nowakowski, R. S. and Caviness, V. S. Jr. (1995). The cell cycle of the pseudostratified ventricular epithelium of the embryonic murine cerebral wall. *J. Neurosci.* 15, 6046-6057. 10.1523/JNEUROSCI.15-09-06046.1995 7666188 PMC6577667

[DEV200076C90] Takashima, Y., Era, T., Nakao, K., Kondo, S., Kasuga, M., Smith, A. G. and Nishikawa, S.-I. (2007). Neuroepithelial cells supply an initial transient wave of MSC differentiation. *Cell* 129, 1377-1388. 10.1016/j.cell.2007.04.028 17604725

[DEV200076C91] Tang, Q.-Y., Zhang, S.-F., Dai, S.-K., Liu, C., Wang, Y.-Y., Du, H.-Z., Teng, Z.-Q. and Liu, C.-M. (2020). UTX regulates human neural differentiation and dendritic morphology by resolving bivalent promoters. *Stem Cell Repo.* 15, 439-453. 10.1016/j.stemcr.2020.06.015 PMC7419705 32679064

[DEV200076C92] Telley, L., Agirman, G., Prados, J., Amberg, N., Fievre, S., Oberst, P., Bartolini, G., Vitali, I., Cadilhac, C., Hippenmeyer, S. et al. (2019). Temporal patterning of apical progenitors and their daughter neurons in the developing neocortex. *Science* 364, eaav2522. 10.1126/science.aav2522 31073041

[DEV200076C93] van Mierlo, G., Veenstra, G. J. C., Vermeulen, M. and Marks, H. (2019). The Complexity of PRC2 Subcomplexes. *Trends Cell Biol.* 29, 660-671. 10.1016/j.tcb.2019.05.004 31178244

[DEV200076C94] Wang, J., Mager, J., Schnedier, E. and Magnuson, T. (2002). The mouse PcG gene eed is required for Hox gene repression and extraembryonic development. *Mamm. Genome* 13, 493-503. 10.1007/s00335-002-2182-7 12370779

[DEV200076C95] Wijayatunge, R., Liu, F., Shpargel, K. B., Wayne, N. J., Chan, U., Boua, J.-V., Magnuson, T. and West, A. E. (2018). The histone demethylase Kdm6b regulates a mature gene expression program in differentiating cerebellar granule neurons. *Mol. Cell. Neurosci.* 87, 4-17. 10.1016/j.mcn.2017.11.005 29254825 PMC5828961

[DEV200076C96] Wolf, F. A., Angerer, P. and Theis, F. J. (2018). SCANPY: large-scale single-cell gene expression data analysis. *Genome Biol.* 19, 15. 10.1186/s13059-017-1382-0 29409532 PMC5802054

[DEV200076C97] Wolf, F. A., Hamey, F. K., Plass, M., Solana, J., Dahlin, J. S., Göttgens, B., Rajewsky, N., Simon, L. and Theis, F. J. (2019). PAGA: graph abstraction reconciles clustering with trajectory inference through a topology preserving map of single cells. *Genome Biol.* 20, 59. 10.1186/s13059-019-1663-x 30890159 PMC6425583

[DEV200076C98] Wu, T., Hu, E., Xu, S., Chen, M., Guo, P., Dai, Z., Feng, T., Zhou, L., Tang, W., Zhan, L. et al. (2021). clusterProfiler 4.0: a universal enrichment tool for interpreting omics data. *Innovation (N Y)* 2, 100141. 10.1016/j.xinn.2021.100141 34557778 PMC8454663

[DEV200076C99] Xie, Y. and Dorsky, R. I. (2017). Development of the hypothalamus: conservation, modification and innovation. *Development* 144, 1588-1599. 10.1242/dev.139055 28465334 PMC5450842

[DEV200076C100] Xie, H., Xu, J., Hsu, J. H., Nguyen, M., Fujiwara, Y., Peng, C. and Orkin, S. H. (2014). Polycomb repressive complex 2 regulates normal hematopoietic stem cell function in a developmental-stage-specific manner. *Cell Stem Cell* 14, 68-80. 10.1016/j.stem.2013.10.001 24239285 PMC3947409

[DEV200076C102] Yaghmaeian Salmani, B., Monedero Cobeta, I., Rakar, J., Bauer, S., Curt, J. R., Starkenberg, A. and Thor, S. (2018). Evolutionarily conserved anterior expansion of the central nervous system promoted by a common PcG-Hox program. *Development* 145, dev160747. 10.1242/dev.160747 29530878

[DEV200076C103] Yee, C. L., Wang, Y., Anderson, S., Ekker, M. and Rubenstein, J. L. R. (2009). Arcuate nucleus expression of NKX2.1 and DLX and lineages expressing these transcription factors in neuropeptide Y(+), proopiomelanocortin(+), and tyrosine hydroxylase(+) neurons in neonatal and adult mice. *J. Comp. Neurol.* 517, 37-50. 10.1002/cne.22132 19711380 PMC3021751

[DEV200076C104] Yoo, S. and Blackshaw, S. (2018). Regulation and function of neurogenesis in the adult mammalian hypothalamus. *Prog. Neurobiol.* 170, 53-66. 10.1016/j.pneurobio.2018.04.001 29631023 PMC6173995

[DEV200076C105] Zainolabidin, N., Kamath, S. P., Thanawalla, A. R. and Chen, A. I. (2017). Distinct activities of Tfap2A and Tfap2B in the specification of GABAergic interneurons in the developing cerebellum. *Front. Mol. Neurosci.* 10, 281. 10.3389/fnmol.2017.00281 28912684 PMC5583517

[DEV200076C106] Zappia, L. and Oshlack, A. (2018). Clustering trees: a visualization for evaluating clusterings at multiple resolutions. *Gigascience* 7, giy083. 10.1093/gigascience/giy083 PMC6057528 30010766

[DEV200076C107] Zeisel, A., Munoz-Manchado, A. B., Codeluppi, S., Lonnerberg, P., La Manno, G., Jureus, A., Marques, S., Munguba, H., He, L., Betsholtz, C. et al. (2015). Brain structure. Cell types in the mouse cortex and hippocampus revealed by single-cell RNA-seq. *Science* 347, 1138-1142. 10.1126/science.aaa1934 25700174

[DEV200076C108] Zeisel, A., Hochgerner, H., Lönnerberg, P., Johnsson, A., Memic, F., van der Zwan, J., Häring, M., Braun, E., Borm, L. E., La Manno, G. et al. (2018). Molecular architecture of the mouse nervous system. *Cell* 174, 999-1014.e22. 10.1016/j.cell.2018.06.021 30096314 PMC6086934

[DEV200076C109] Zhang, Q., Zhang, L., Huang, Y., Ma, P., Mao, B., Ding, Y.-Q. and Song, N.-N. (2021a). Satb2 regulates the development of dopaminergic neurons in the arcuate nucleus by Dlx1. *Cell Death Dis.* 12, 879. 10.1038/s41419-021-04175-9 34564702 PMC8464595

[DEV200076C110] Zhang, Y. H., Xu, M., Shi, X., Sun, X. L., Mu, W., Wu, H., Wang, J., Li, S., Su, P., Gong, L. et al. (2021b). Cascade diversification directs generation of neuronal diversity in the hypothalamus. *Cell Stem Cell* 28, 1483-1499.e8. 10.1016/j.stem.2021.03.020 33887179

[DEV200076C111] Zhou, X., Zhong, S., Peng, H., Liu, J., Ding, W., Sun, L., Ma, Q., Liu, Z., Chen, R., Wu, Q. et al. (2020). Cellular and molecular properties of neural progenitors in the developing mammalian hypothalamus. *Nat. Commun.* 11, 4063. 10.1038/s41467-020-17890-2 32792525 PMC7426815

