# Supplementary material for: Selective requirement for polycomb repressor complex 2 in the generation of specific hypothalamic neuronal subtypes
Source: Development. 2022 Mar 7;149(5):dev200076. doi: 10.1242/dev.200076 (PMC8959139; doi:10.1242/dev.200076)
Supplement: Supplementary information [file develop-149-200076-s1.pdf]

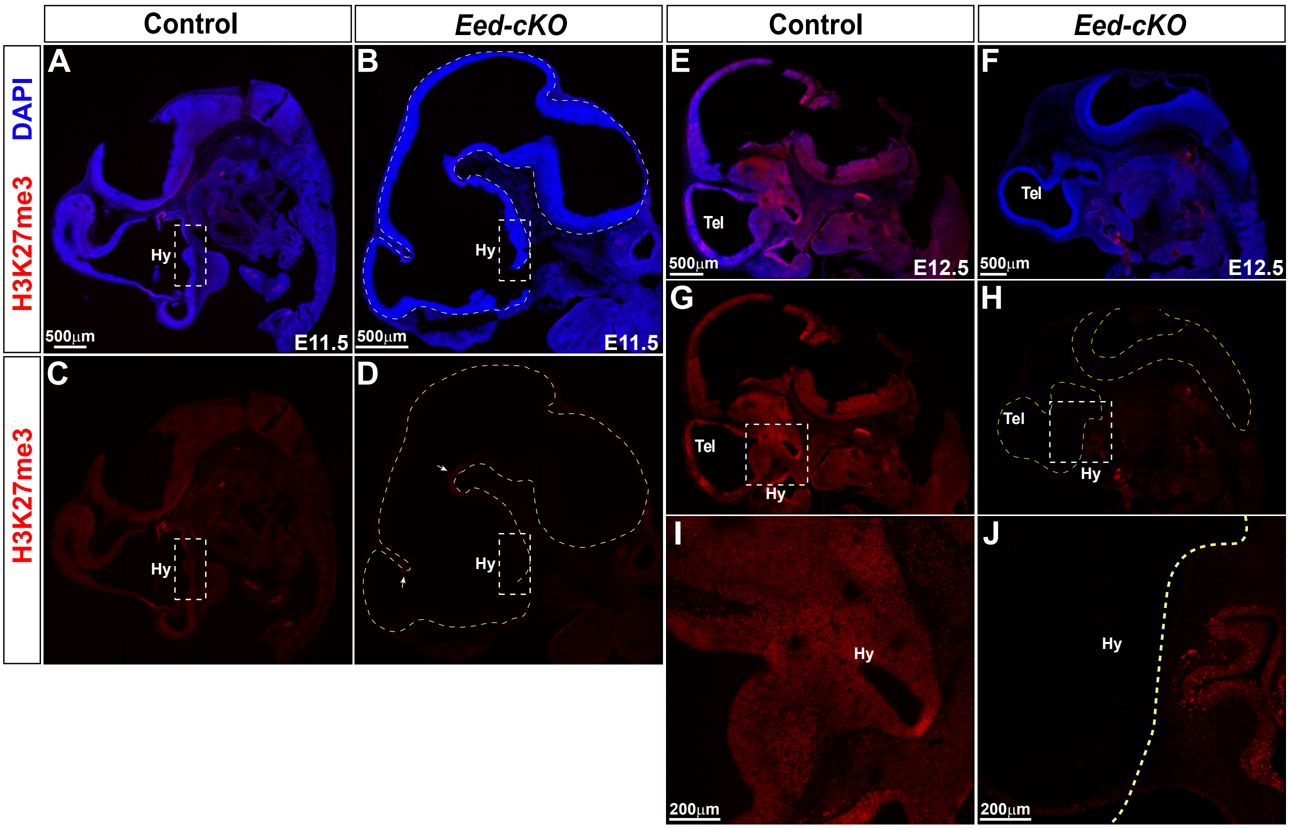

Fig. S1. ***Eed-cKO*** **mutants display loss of H3K27me3.**
(A-D) Sagittal sections of E11.5 control and *Eed-cKO* embryos, stained with DAPI and immunostained for H3K27me3. White dashed line in (D) delineates the CNS in the *Eed-cKO*. In control, H3K27me3 staining is observed throughout the CNS. In *Eed-cKO*, staining is below detection in most parts of the CNS, including in the hypothalamus. A weak signal is apparent in the *Eed-cKO* mutants at the boundary between Tel- and Diencephalon, as well as at the boundary between the Diencephalon and Mid-brain (arrows in D). (E-J) Sagittal sections of E12.5 control and *Eed-cKO* embryos, stained with DAPI and H3K27me3. White, dashed square insets in (G-H) indicate hypothalamic regions in control and *Eed-cKO* magnified in (I) and (J) respectively. Yellow dashed line in H and J delineates the CNS, including the hypothalamic region. In control, H3K27me3 staining is observed throughout the CNS. In *Eed-cKO*, staining is below detection in all parts of the CNS, including in the hypothalamus.

Development • Supplementary information

Eed-cKO mutants display reduced proliferation

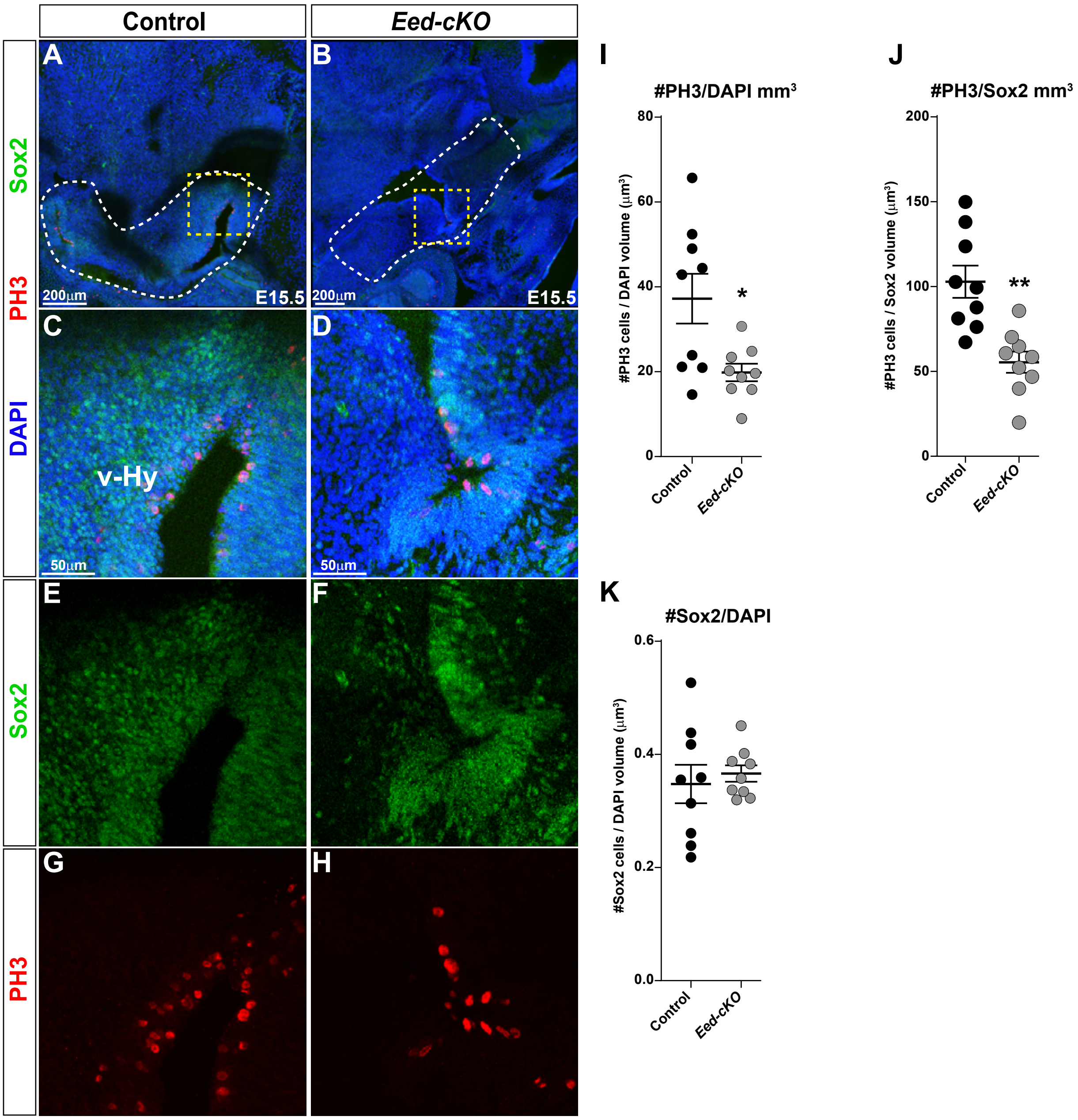

Fig. S2. *Eed-cKO* mutants display reduced proliferation in the hypothalamus.
(A-H) Staining for Sox2, PH3 and DAPI in sagittal sections of E15.5 embryos, in control and *Eed-cKO*. (A-B) White dashed lines delineate the hypothalamic region used for proliferation analysis. Dashed square insets delineate regions magnified in (C-H). (I, J) Quantification of proliferation in hypothalamic tissue of control vs *Eed-cKO*, at E15.5, plotted as PH3+ cells per mm^3 of DAPI and Sox2 signals respectively, shows reduced proliferation in *Eed-cKO*. (K) Ratios of Sox2/DAPI signal did not change between hypothalamic tissues of control and *Eed-cKO* embryos at E15.5. Student's t-test; mean +/- SEM; n= 3 embryos per genotype, n=9 sections; 3 sections per embryo, per genotype (for source data, see Table S7).

Development • Supplementary information

Technical Filtering of scRNA-seq data

Technical Filtering

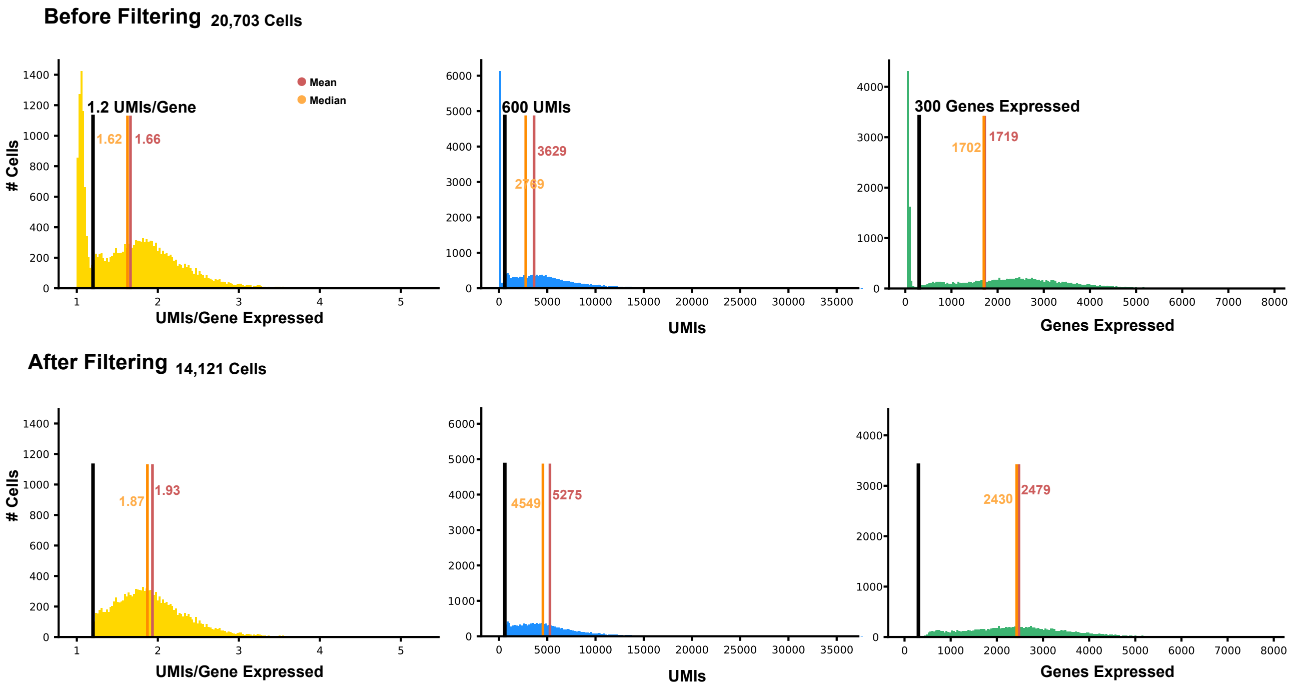

Fig. S3. Technical filtering of E18.5 scRNA-seq data.
Technical filtering of E18.5 scRNA-seq data depicts histograms of the Unique Molecular Identifiers (UMIs) expressed per gene, the total UMIs, and the genes expressed across the cells both before (top) and after (bottom) quality control, respectively. The black, red, and orange vertical lines indicate the cut offs used for quality control, the mean for each quality metric, and the median for each quality metric, respectively.

Development • Supplementary information

Supplemental Figure 4

Gene Analysis in *Eed-cKO* Mutants

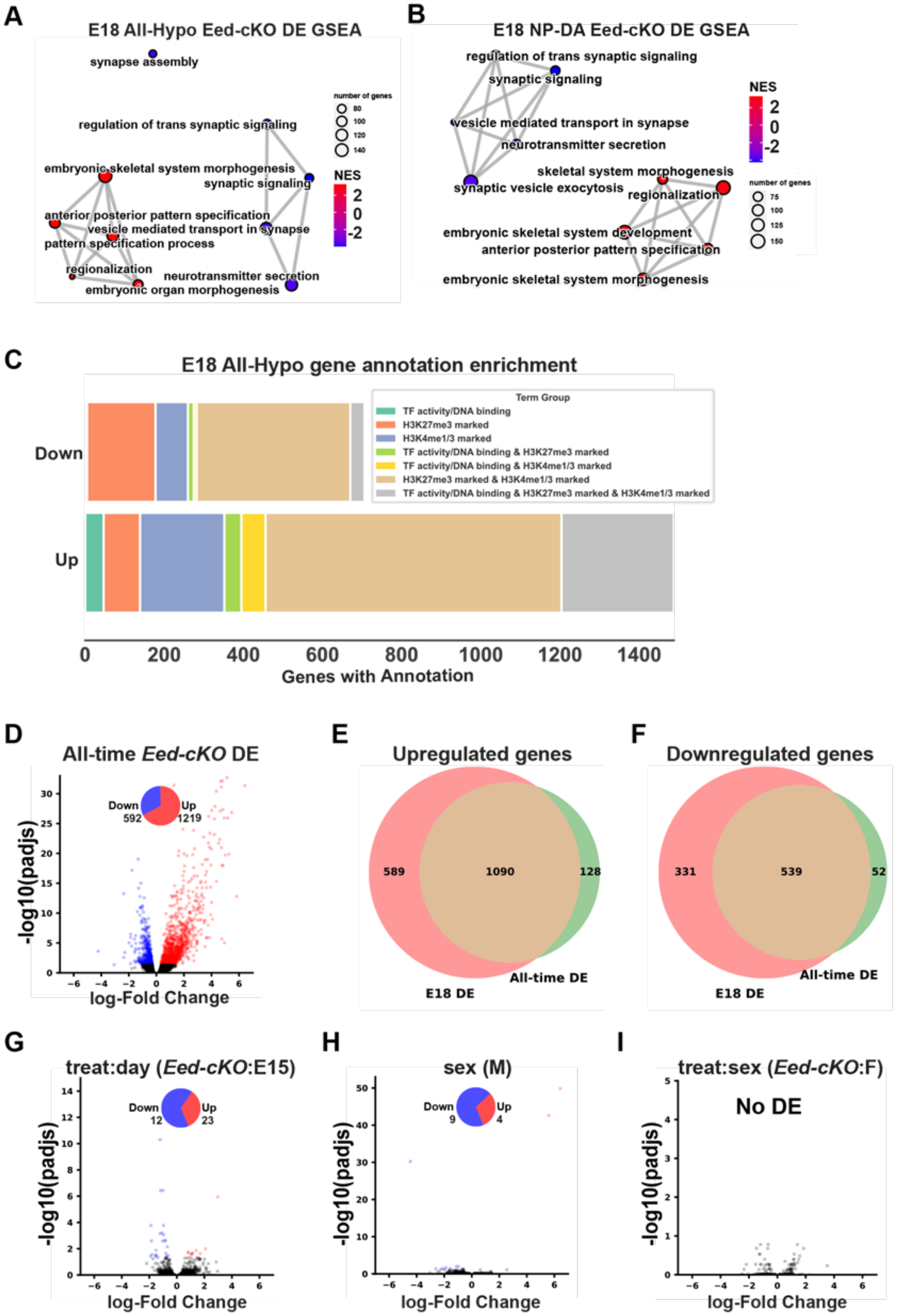

Fig. S4. GO analysis of scRNA-seq data.
(A-B) Gene Set Enrichment Analysis (GSEA) of All-Hypo (A) and NP-DA cells (B) for DE genes affected in *Eed-cKO* (adjusted p-value *<0.05).* (C) GO analysis of All-Hypo cells. The total number of gene sets ("Terms") grouped as "TF activity/DNA binding" if they mention "binding" or "transcription" in the term name, or "H3K27me3" or "H3K4me3" if these epigenetic marks were mentioned in the term name; the remaining terms are grouped as "Other". The epigenetic terms indicate genes marked by the respective modification in a biological tissue sample in the *ENCyclopaedia of DNA Elements* (ENCODE) (see Table S3 for details). (D) Vulcano plot showing DE genes in *Eed-cKO* mutants at E15.5 (see Table S3 for details). (E-F) Overlap between DE genes at E18.5 and DE genes at all stages analysed, showing extensive overlap between both up- and downregulated genes at the different stages. (G) Vulcano plot showing DE genes in *Eed-cKO* mutants at E15.5 when compared to DE genes at other stages (see Table S3 for details), showing that very few genes are DE specifically at E15.5. (H) Vulcano plot showing DE genes in males versus females at all stages (see Table S3 for details). (I) Vulcano plot showing DE genes in males versus females in control versus in *Eed-cKO* mutants at E15.5 (see Table S3 for details), showing that all sexually dimorphic genes are still dimorphic in *Eed-cKO*.

Development • Supplementary information

Eed-cKO mutants display ectopic Gfap expression

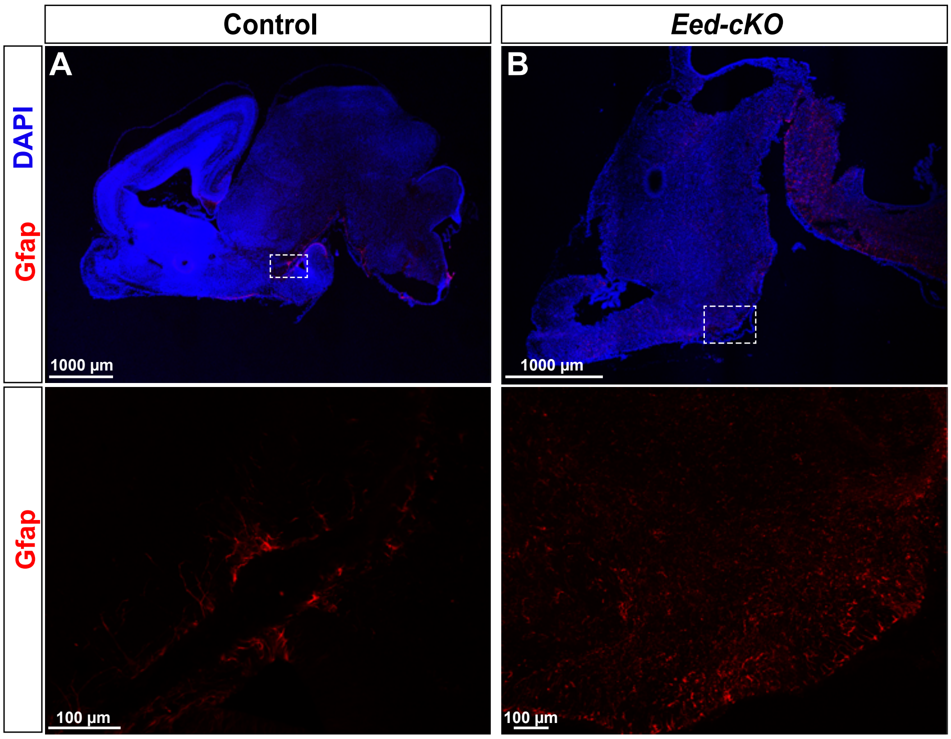

Fig. S5. *Eed-cKO* mutants display ectopic Gfap expression.
(A-B) Staining for DAPI and Gfap in sagittal sections of E18.5 brains, in control and *Eed-cKO*. Dashed, square insets delineate the ventral hypothalamus. In control, Gfap expression is primarily observed along the epithelium. In *Eed-cKO* mutants, Gfap expression is increased and expands into the hypothalamus.

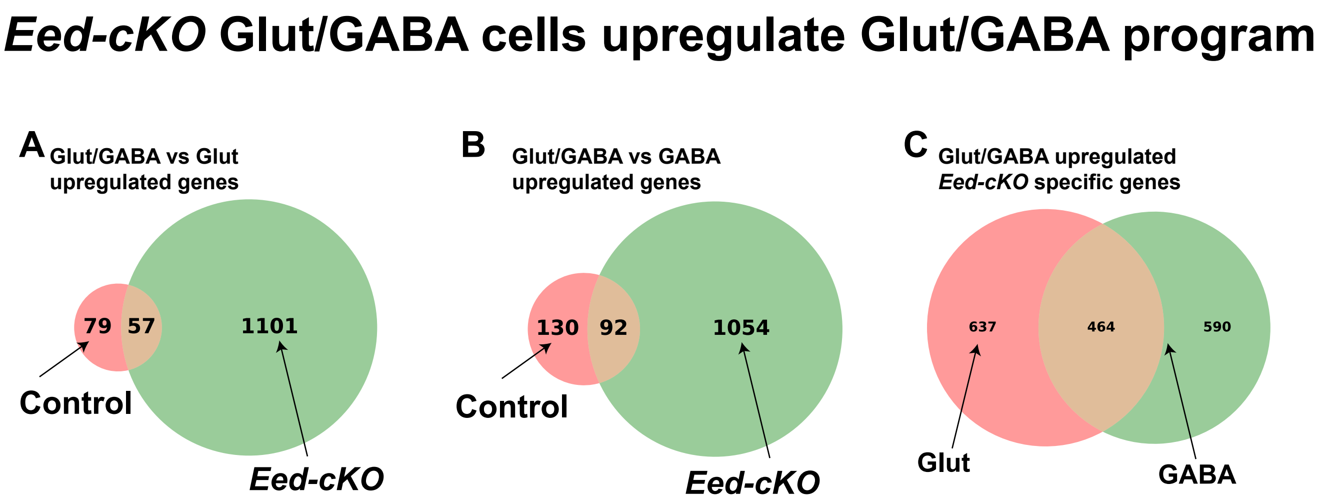

Fig. S6. ***Eed-cKO*** **Glut/GABA cells upregulate partial wild type Glut/GABA program.** (A) DE genes (upregulated) when comparing Glut/GABA cells versus Glut cells in control (pink) and *Eed-cKO* (green), and the overlap (orange), revealing that the extra Glut/GABA cells observed in *Eed-cKO* express a gene profile that only partly overlaps with control Glut/GABA cells. (B) The equivalent to A, except comparing the upregulated DE genes in Glut/GABA cells against GABA cells identified within each condition; again, indicating a high overlap with control but disproportionate effect of *Eed-cKO* on the glut-GABA cells. (C) Comparison between the *Eed-cKO* specific genes in Glut/GABA cells when comparing them with Glut and GABA cells (i.e., overlap of the green circles from A and B).

Increased Glut/GABA in NP neurons in *Eed-cKO* mutants

A **Control**

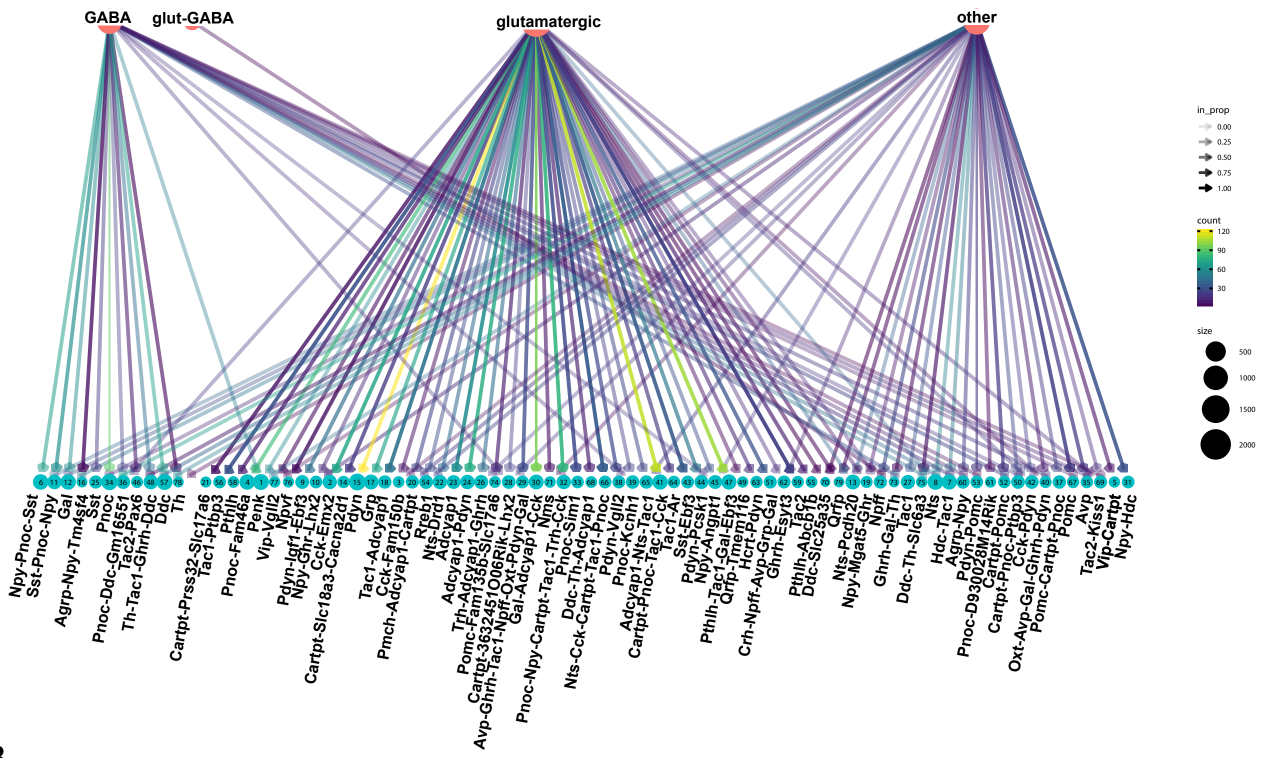

B ***Eed-cKO***

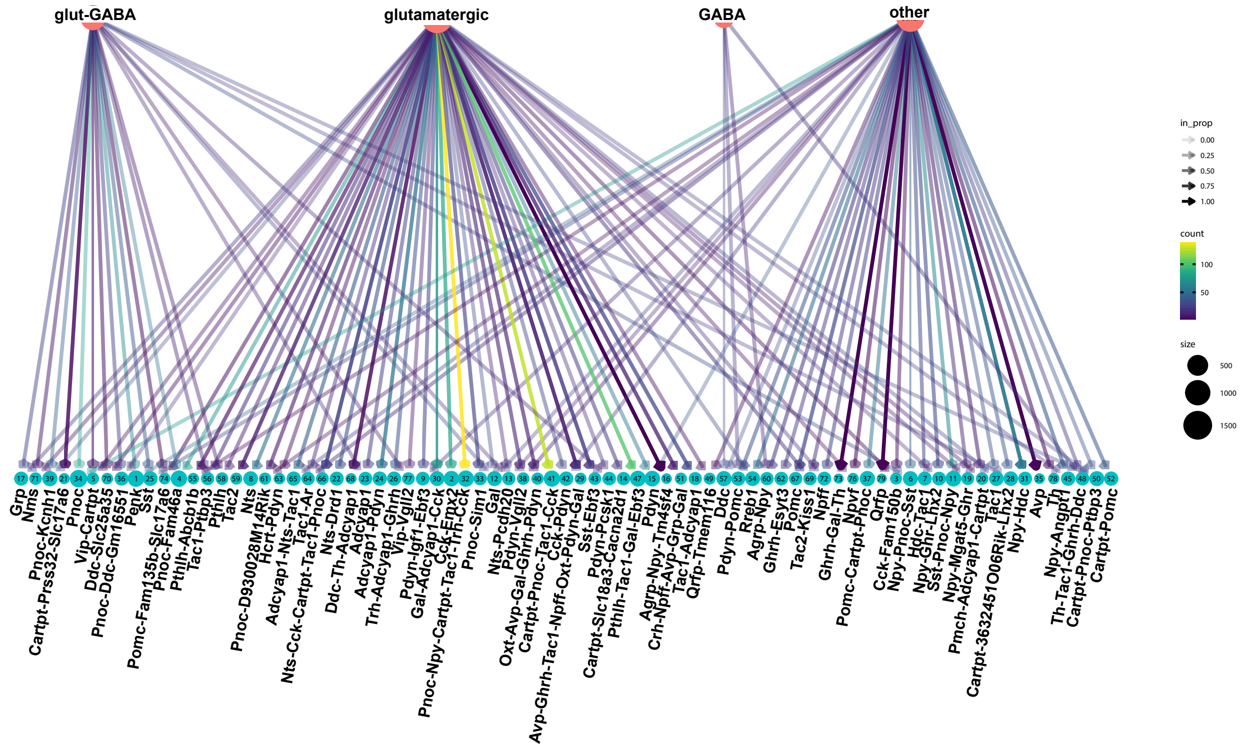

Development • Supplementary information

Fig. S7. Increased Glut/GABA in NP neurons in *Eed-cKO* mutants.
(A-B) Clustree representation displaying the distribution of fast neurotransmitter status in the 79 NP-DA E18.5 clusters; GABA (*Slc32a1*), Glut (*Slc17a6*), Glut/GABA (*Slc32a1* and *Slc17a6*) and “other” (no *Slc32a1* or *Slc17a6*). Arrows from the top row to the bottom row indicate where >25% of the cells in the NP-DA cluster are of the respective neurotransmitter status. Arrow opacity indicates the proportion of cells in the NP-DA cluster which are also in the respective fast neurotransmitter group. Arrow colors indicate total no. of cells which have the respective neurotransmitter and NP-DA cluster status. Point sizes indicated the total number of cells in each group. (A) In control, Glut/GABA expression is primarily restricted to *Npff* cells. (B) In *Eed-cKO* mutants, Glut/GABA expression spreads into several different neuropeptide cells.

Ectopic expression of posterior genes in *Eed-cKO*

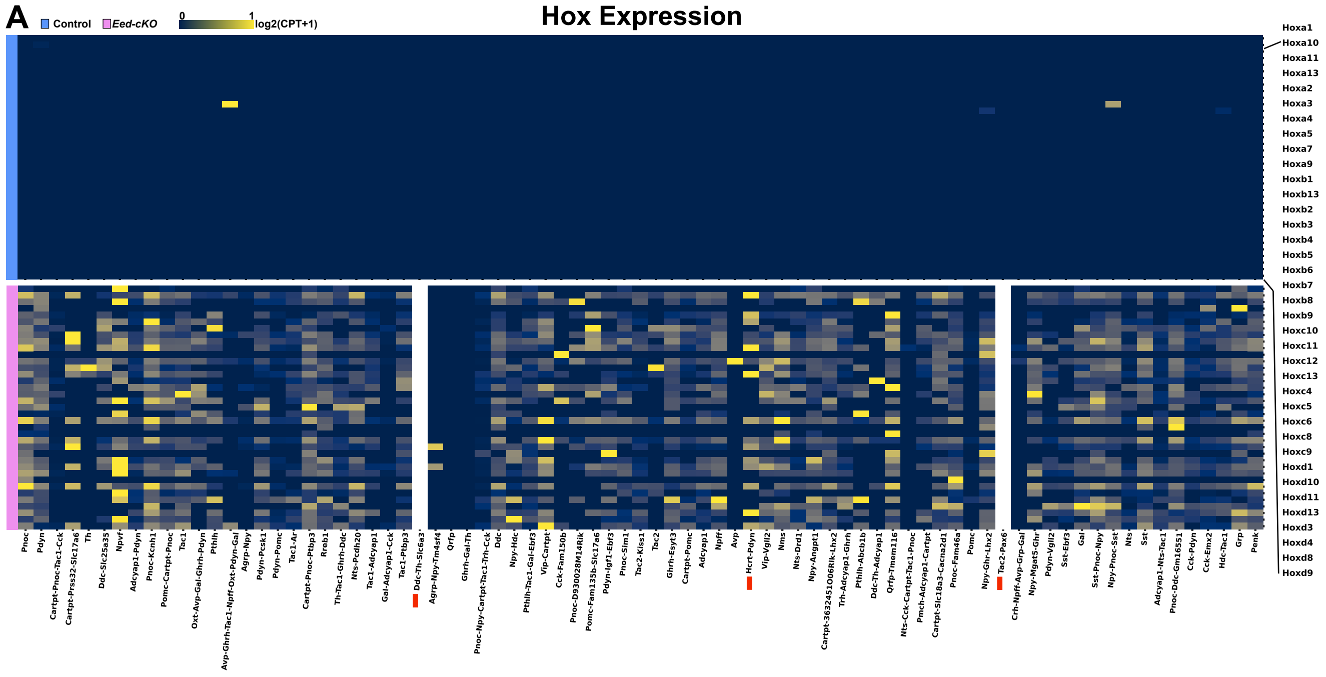

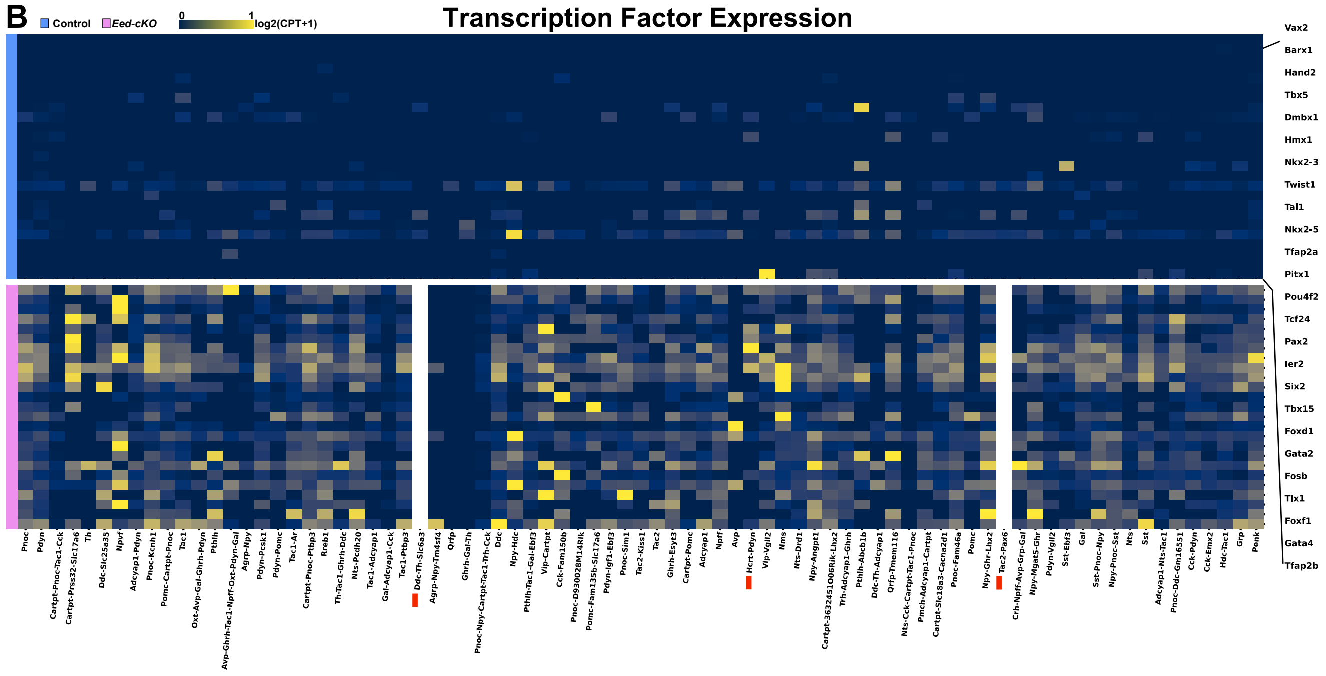

Development • Supplementary information

Fig. S8. ***Eed-cKO* mutants display Hox and posterior TF expression in the hypothalamus.** (A-B) Heatmap displaying average gene expression of the Hox and posterior TF genes in each UMAP cell cluster, in control (blue) and *Eed-cKO* (pink). Gene expression is measured in log2(CPT+1), scaled between 0 and 1 along each row. The *Ddc-Th-Slc6a3*, *Tac2* and *Hcrt* clusters are absent in *Eed-cKO,* and hence a column of 0 expression was depicted.

Supplemental Figure 9

Eed is not necessary for most NP-DA fates

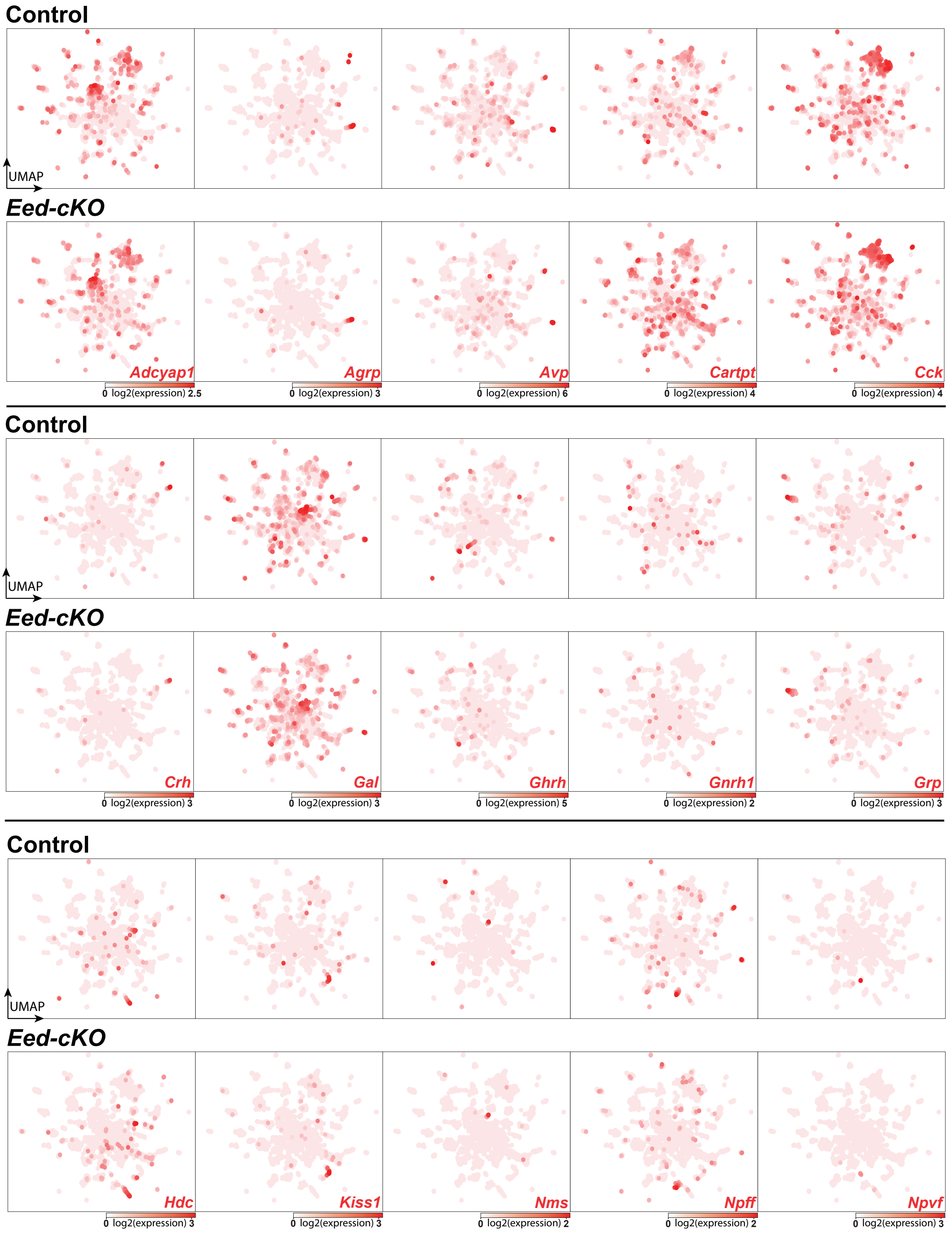

Fig. S9. ***Eed-cKO*** **is not necessary for most NP-DA cell fates.**
UMAP embedding of E18.5 NP-DA cells, based upon 122 DE genes (Table S1), showing expression of neuropeptide genes, in control and *Eed-cKO* cells.

Development • Supplementary information

Supplemental Figure 10

Eed is not necessary for most NP-DA fates

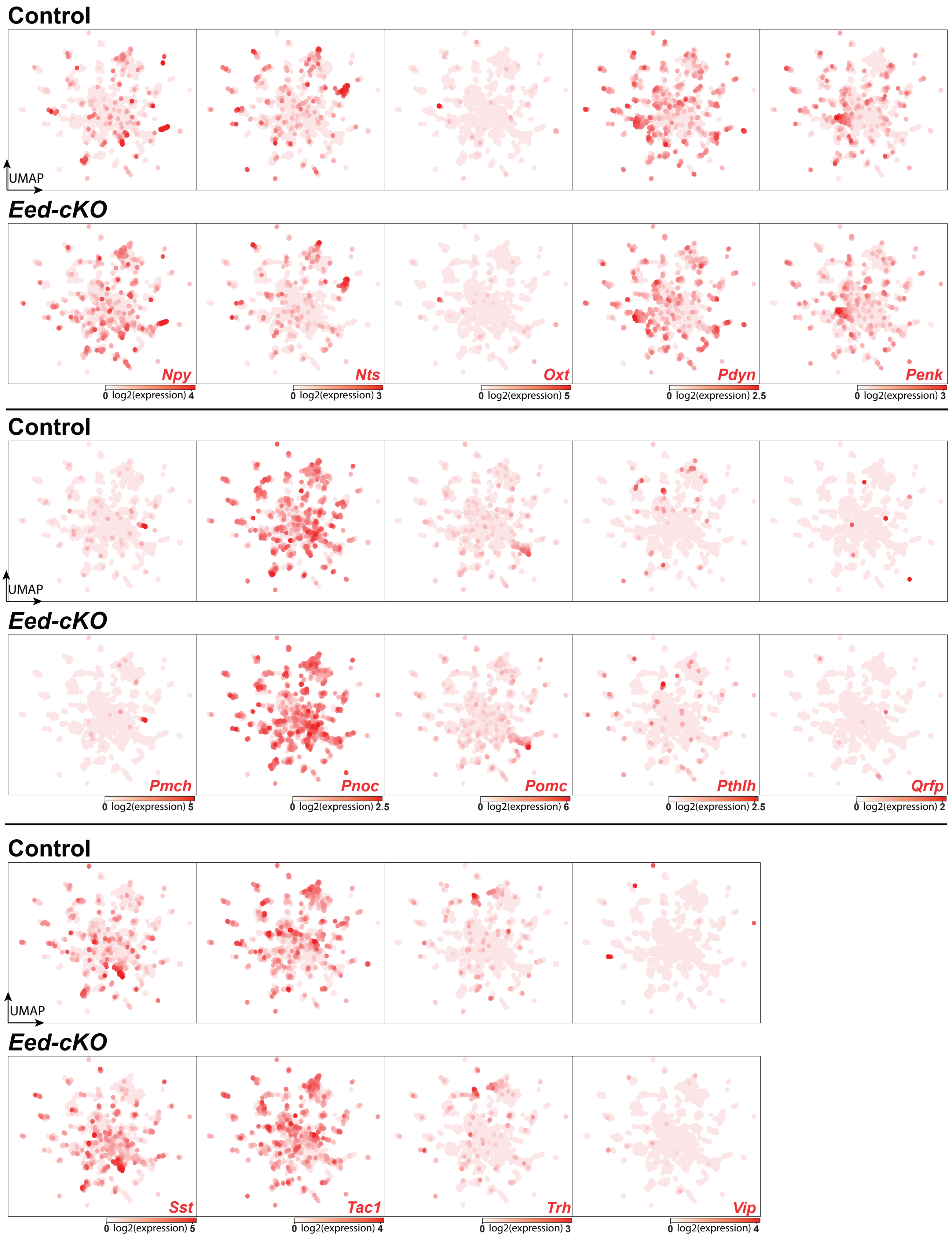

Fig. S10. ***Eed-cKO* is not necessary for most NP-DA cell fates.**
UMAP embedding of E18.5 NP-DA cells, based upon 122 DE genes (Table S1), showing expression of neuropeptide genes, in control and *Eed-cKO* cells.

Development • Supplementary information

Eed is not critical for Avp neurons

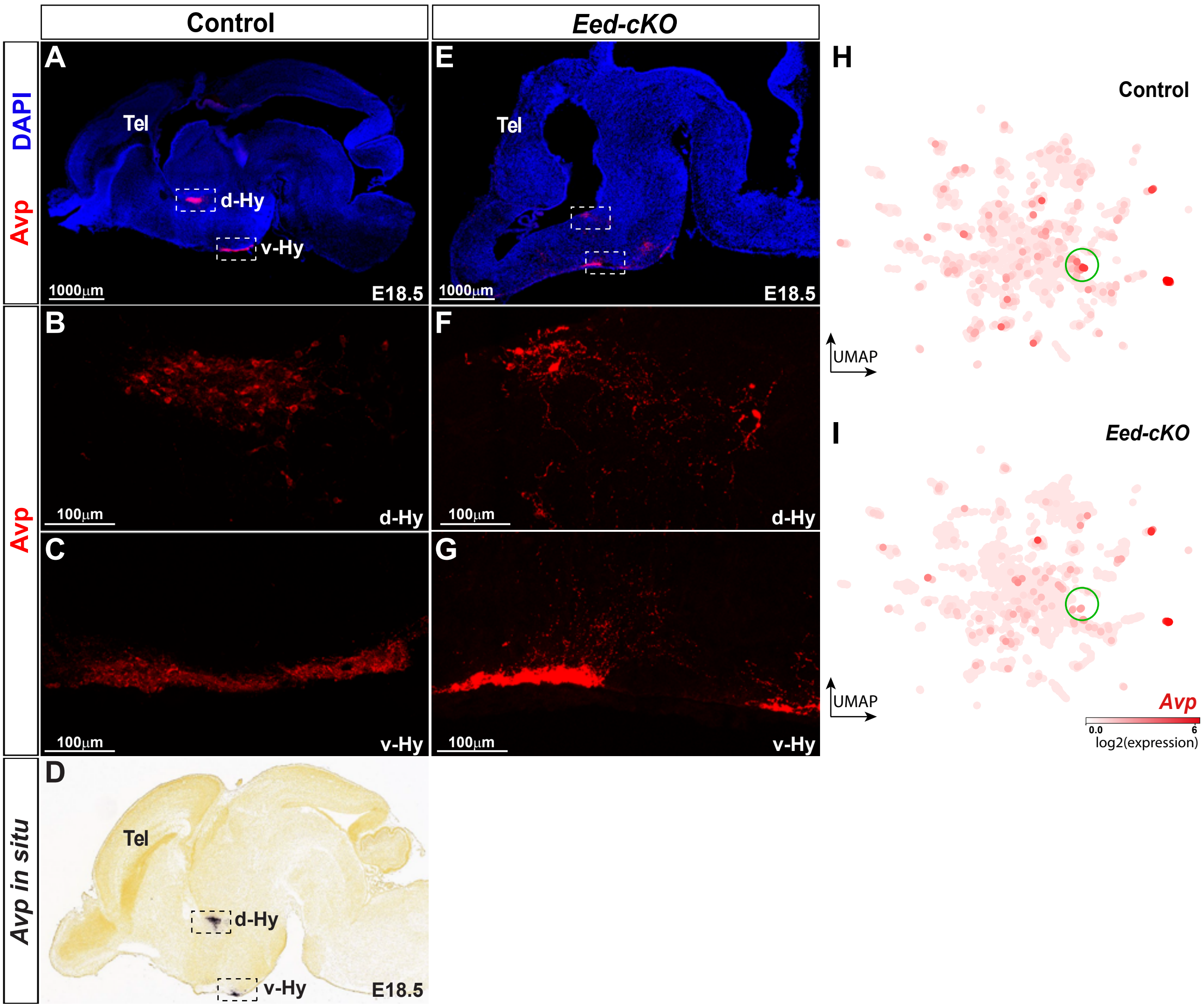

Fig. S11. ***Eed*** **is not critical for the generation of Avp neurons.**
(A-C, E-G) Staining for Avp and DAPI in sagittal sections of E18.5 control and *Eed-cKO* mutant brains. Dashed, square insets delineate Avp cells in dorsal and ventral hypothalamic regions. (C-F) Avp+ cells in dorsal (d-Hy) and ventral (v-Hy) regions of hypothalamus in control and *Eed-cKO* embryos. The organization and clustering of v-Hy region Avp cells appear to be disrupted in *Eed-cKO*. (D) *In situ* hybridisation of *Avp* in cross-section of E18.5 mouse embryo (image from Allen Brain Atlas) (Thompson et al., 2014). (H-I) UMAP embedding of E18.5 hypothalamic scRNA-seq NP-DA cells, with each cell coloured according to the expression level of *Avp* in control and *Eed-cKO*. Scale bar; 1000 µm in (A, B), 100 µm in (C-F).

Development • Supplementary information

Supplemental Figure 12

Quality control and analysis of early time points

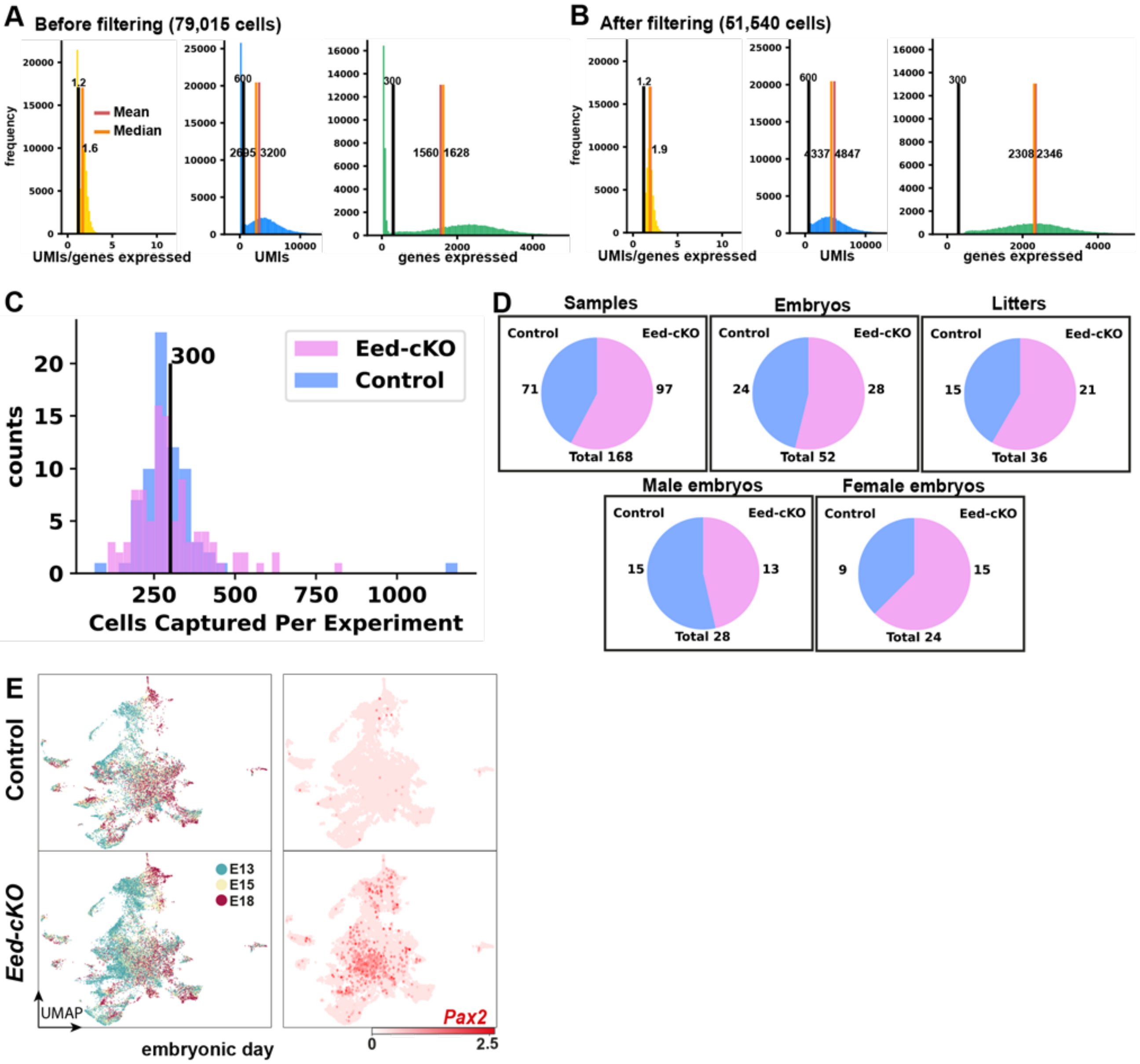

Development • Supplementary information

Fig. S12. Technical filtering and analysis of E13.5 and E15.5 scRNA-seq data.
(A-B) Technical filtering depicts histograms of the Unique Molecular Identifiers (UMIs) expressed per gene, the total UMIs, and the genes expressed across the cells both before and after quality control, respectively. The black, red, and orange vertical lines indicate the cut offs used for quality control, the mean for each quality metric, and the median for each quality metric, respectively. (C) Average high-quality cell yields/sample. (D) Litters, embryos and samples analysed for control and *Eed-cKO*. (E) UMAP embedding of E13.5, E15.5 and E18.5 cells, showing a similar temporal profile of development in the hypothalamus in *Eed-cKO* mutants. However, *Pax2* expression is upregulated.

Development • Supplementary information

Eed-cKO Glut/GABA increase is not due to doublets

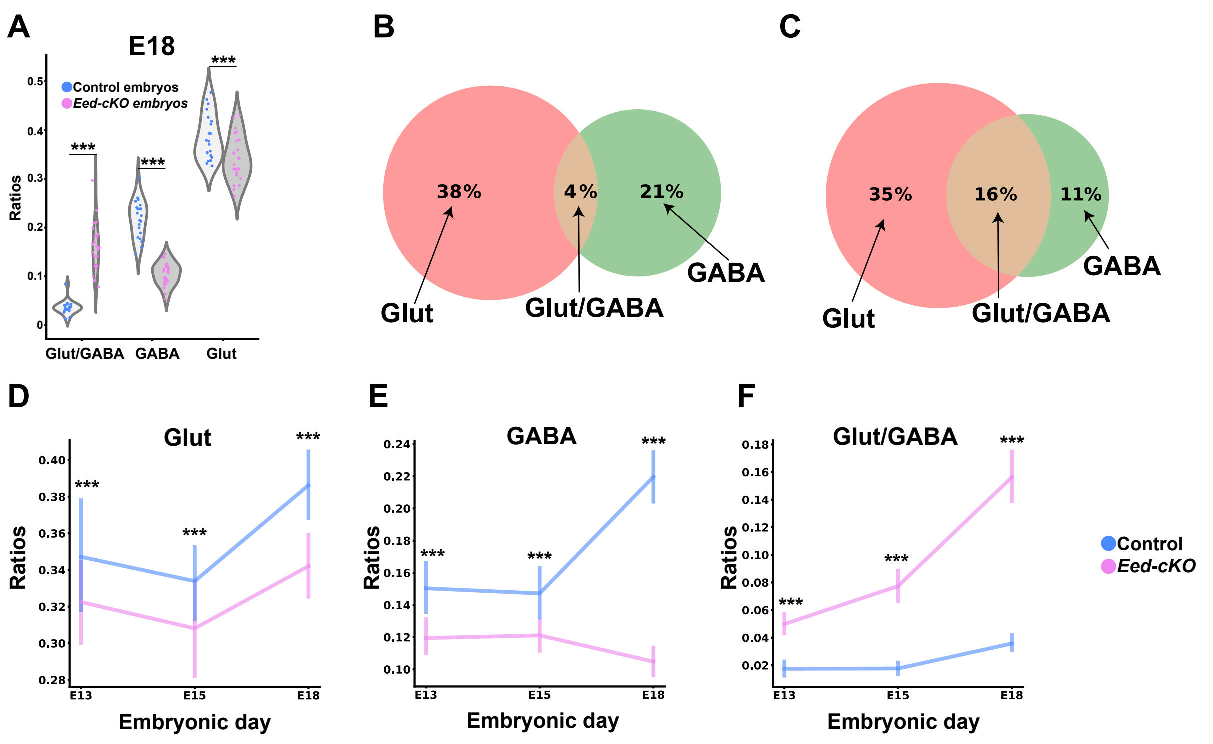

Fig. S13. Doublet filtering does not nullify the increased Glut/GABA cells effect in *Eed-cKO*.

(A-F) Quantification of Glut, GABA and Glut/GABA cell ratios at E13.5, E15.5 and E18.5 in control and *Eed-cKO* mutants after doublet removal. The enrichment of Glut/GABA cells in *Eed-cKO* is evident also after doublet removal.

Development • Supplementary information

Eed-cKO shows normal tanycyte/ependymal trajectories

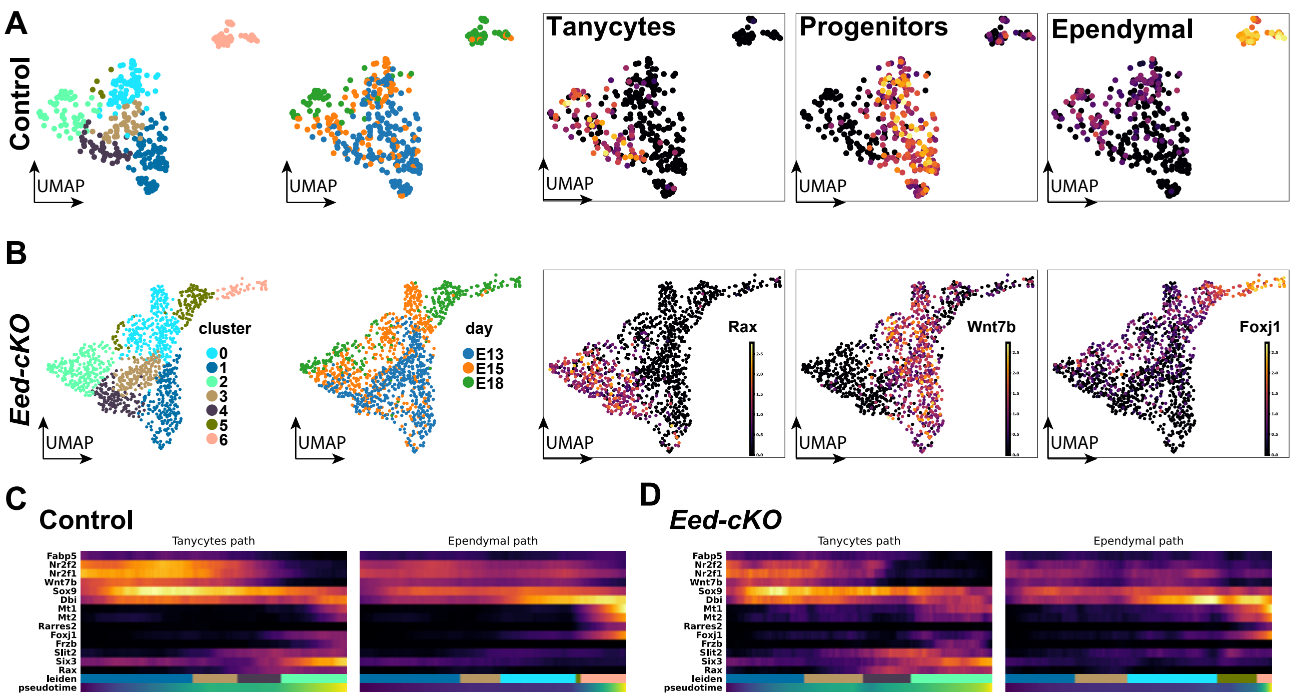

Fig. S14. ***Eed-cKO* mutants display normal tanycyte and ependymal trajectories.** (A-B) UMAPs illustrating the branching trajectory of tanycyte (*Rax*) and ependymal (*Foxj1*) cells from a common progenitor pool (*Wnt7b*), in control and *Eed-cKO*. From left-to-right, UMAPs display Leiden clustering of cells, the embryonic time point of mice from which the cells were sampled, and the marker gene expression of *Rax*, *Wnt7b*, and *Foxj1*. In both control and *Eed-cKO* there is an equivalent trajectory of *Wnt7b* progenitor cells branching to create *Rax*+ tanycytes and *Foxj1*+ ependymal cells. (C-D) Heatmaps displaying gene expression trends of genes previously correlated with tanycyte development (Kim et al., 2020) along the pseudotime of cells from Leiden cluster 1 to 3, 4, and then the cluster 2 as the tanycyte path. Psuedotime for the ependymal path follows from cluster 1 to 3, then 0 and 5 with the mature ependymal cells falling within cluster 6. The same developmental gene expression trends are observed between control and *Eed-cKO* mice in tanycyte/ependymal cell type development.

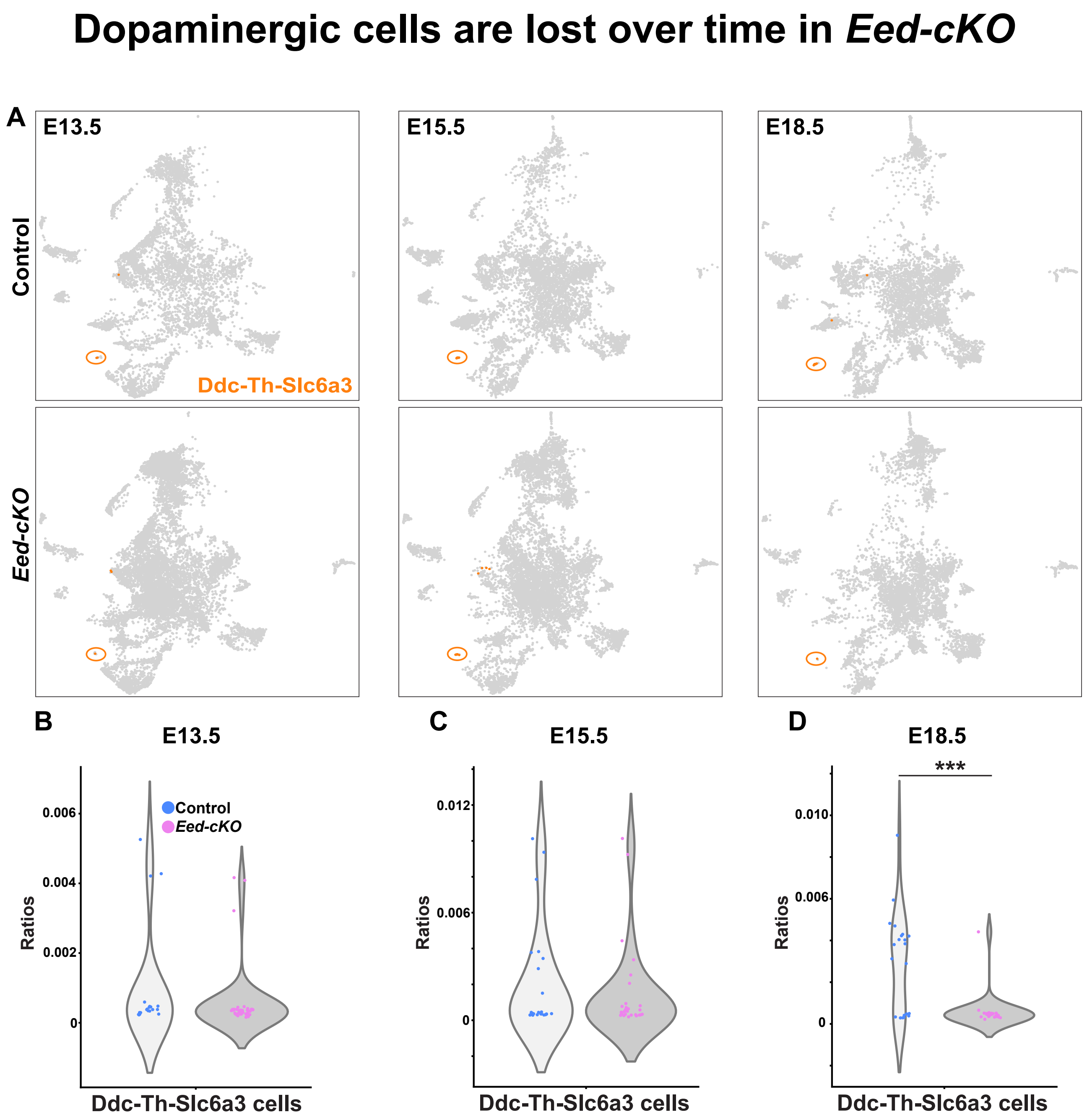

Fig. S15. Dopaminergic cells are lost over time in *Eed-cKO* mutants.
(A) UMAPs displaying *Ddc-Th-Slc6a3* triple expressing cells, in control and *Eed-cKO*. One major cluster is evident in both control and mutants, at E13.5 and E15.5, while this cluster is severely reduced in size at E18.5 in the mutant. (B-D) Quantification *Ddc-Th-Slc6a3* triple expressing cells, in control and *Eed-cKO*. At E13.5 and E15.5, there is no significant reduction of the proportion of triple expressing cells, while at E18.5 these cells are severely reduced in *Eed-cKO*.

Hcrt and *Tac2-Pax6* cells are absent in *Eed-cKO* mutants

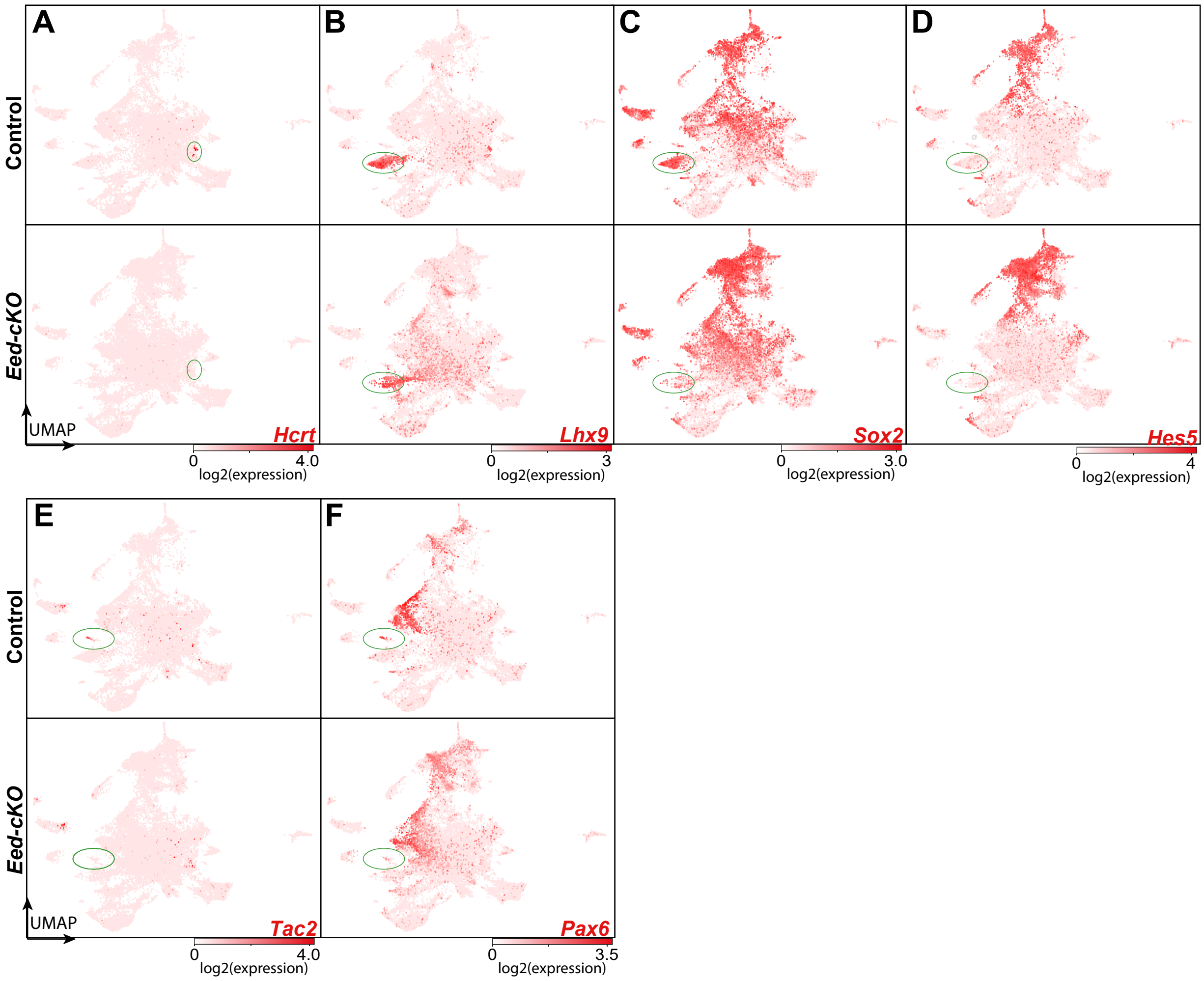

Fig. S16. **Expression of Hcrt and Tac2.**
(A-F) UMAPs displaying *Hcrt*, *Lhx9*, *Sox2*, *Hes5*, *Tac2* and *Pax6* expressing cells, in control and *Eed-cKO*, combining cells from E13.5, E15.5 and E18.5. (A) One major *Hcrt* cluster is evident in control but largely lost in the mutant. (B-D) A branch of *Lhx9*+, *Sox2*+ and *Hes5*- progenitor cells is evident in control but severely reduced in the mutant. (E) One major *Tac2* cluster is evident in control but largely lost in the mutant. (F) This *Tac2* cluster also expresses *Pax6*, which is also severely reduced in the mutant.

Table S1. Gene lists used for UMAP analysis.

Click here to download Table S1

Table S2. Differential expression in the All-Hypo and NP-DA cells.

Click here to download Table S2

Table S3. GSEA/ENCODE peak enrichment analysis of DE genes in the All-Hypo cells scRNA-seq data.

Click here to download Table S3

Table S4. Differentially expressed genes comparison between glut-GABA cells and glutamatergic/GABAergic cells within control or Eed-cKO.

Click here to download Table S4

Table S5. NP-DA cell type counts stratified by different annotations.

Click here to download Table S5

Table S6. DE genes per NP-DA UMAP cluster.

Click here to download Table S6

Table S7. Temporal onset of NP-DA genes.

Click here to download Table S7

Table S8. Source Data for Figure S2.

Click here to download Table S8

Table S9. Glut/GABA cell type counts stratified by different annotations.

Click here to download Table S9

Table S10. Differential abundance of glut-GABA cells identified between control and Eed-cKO embryos with and without doublet removal and across developmental time points.

Click here to download Table S10

Table S11. Lhx9 and Glut-GABA subpopulations compared between Eed-cKO and control.

Click here to download Table S11

Table S12. Marker genes used for broad labelling of early time points & confusion matrix from logistic regression classification.

Click here to download Table S12

Table S13. Marker genes used for broad labelling of early time points & confusion matrix from logistic regression classification.

Click here to download Table S13
